# Global quantitative proteomics reveal up-regulation of endoplasmic reticulum stress response proteins upon depletion of eIF5A in HeLa cells

**DOI:** 10.1038/srep25795

**Published:** 2016-05-16

**Authors:** Ajeet Mandal, Swati Mandal, Myung Hee Park

**Affiliations:** 1 Molecular and Cellular Biochemistry Section, Oral and Pharyngeal Cancer Branch, National Institute of Dental and Craniofacial Research, National Institutes of Health, Bldg.30 Rm. 3A300, Bethesda, MD 20892, USA

## Abstract

The eukaryotic translation factor, eIF5A, is a translation factor essential for protein synthesis, cell growth and animal development. By use of a adenoviral eIF5A shRNA, we have achieved an effective depletion of eIF5A in HeLa cells and undertook *in vivo* comprehensive proteomic analyses to examine the effects of eIF5A depletion on the total proteome and to identify cellular pathways influenced by eIF5A. The proteome of HeLa cells transduced with eIF5A shRNA was compared with that of scramble shRNA-transduced counterpart by the iTRAQ method. We identified 972 proteins consistently detected in three iTRAQ experiments and 104 proteins with significantly altered levels (protein ratio ≥1.5 or ≤0.66, p-value ≤0.05) at 72 h and/or 96 h of Ad-eIF5A-shRNA transduction. The altered expression levels of key pathway proteins were validated by western blotting. Integration of functional ontology with expression data of the 104 proteins revealed specific biological processes that are prominently up- or down-regulated. Heatmap analysis and Cytoscape visualization of biological networks identified protein folding as the major cellular process affected by depletion of eIF5A. Our unbiased, quantitative, proteomic data demonstrate that the depletion of eIF5A leads to endoplasmic reticulum stress, an unfolded protein response and up-regulation of chaperone expression in HeLa cells.

The highly conserved eukaryotic translation factor eIF5A is strictly indispensable for the survival of eukaryotic cells. eIF5A was initially isolated from rabbit reticulocyte lysates^1^ as a factor that stimulates methionyl puromycin synthesis, a model assay for the first peptide bond formation. Yet, the true physiological function and the mechanism of action of eIF5A *in vivo* have remained elusive for decades^2^,^3^,^4^. eIF5A undergoes a unique posttranslational modification that converts a specific lysine residue to an unusual amino acid, hypusine [N^ε^-(4-amino-2-hydroxybutyl)lysine]^5^,^6^. This hypusine modification is required for eIF5A activity and occurs by way of two enzymatic steps [see review^7^] involving deoxyhypusine synthase (DHS) and deoxyhypusine hydroxylase (DOHH).

A potential function of eIF5A has been inferred from studies of its bacterial ortholog, elongation factor P (EF-P). EF-P stimulates N-formyl-methionyl-puromycin synthesis *in vitro*^8^ and displays similarity to eIF5A^2^,^3^,^9^ and its archaeal homolog, aIF5A, in amino acid sequence and in crystal structure. EF-P of certain bacterial strains contains a β-lysyl-lysine residue^10^,^11^ at a site corresponding to hypusine in eIF5A. The crystal structure of *Thermus thermophilus* EF-P bound to 70S ribosome^12^ revealed the binding of EF-P to the ribosome between the peptidyl tRNA and the exit tRNA sites, suggesting its role in proper positioning of N-formyl-methionyl-tRNA(i) for the formation of the first peptide bond. EF-P has been recently identified as a factor that relieves ribosome stalling at consecutive prolines during translation elongation^13^,^14^. It enhanced the synthesis of polyproline peptides and proteins containing *e.g.* PPP (three consecutive prolines, Pro-Pro-Pro) and PPG (Pro-Pro-Gly) motifs *in vitro* and in bacterial cells. This proposed mechanism of EF-P is well supported by extensive analyses, including proteomics and ribosome profiling using the mutant strains deleted of *efp* or its modifying enzyme genes^15^,^16^,^17^.

With respect to the mechanism of eIF5A in translation, a relatively small inhibition of protein synthesis upon depletion of eIF5A in a *S. cerevisiae* mutant strain^18^ suggested that eIF5A is not a general translation factor, but a specific factor required for the translation of a subset of mRNAs. Polysome profiles of *S. cerevisiae* eIF5A temperature sensitive mutants provided evidence that eIF5A has distinct effects on translation elongation^19^,^20^. Based on the structural analogy of EF-P and eIF5A, the function of the two proteins has been assumed to be conserved^2^,^3^,^9^. Indeed, a recent report has provided evidence for a potentially critical role of eIF5A in translation of polyproline motifs^21^ in *S. cerevisiae*. However, it is still unclear whether elongation of polyprolyl motifs is the primary function of eIF5A^4^,^9^ and how eIF5A deficiency results in pleotropic phenotypes reported in yeast eIF5A mutant strains, including defects in mRNA turnover^22^, cell polarity, actin dynamics, cell wall integrity, cell cycle progression^23^,^24^ and cotranslational translocation of proteins into the ER^25^.

In mammalian cells, eIF5A function has been investigated using inhibitors of DHS and DOHH^7^, eIF5A siRNA, a stable shRNA cell line or by overexpression of eIF5A or its isoform. These studies uncover a complex picture of eIF5A action in different biological systems and have led to diverse proposed functions of eIF5A, in cellular processes including cell cycle progression^26^, nucleo-cytoplasmic transport, HIV1 replication^27^,^28^ apoptosis and tumorigenesis^29^ stress granule formation^30^, inflammation and diabetes^31^ and proliferation and migration and metastasis of cancer cells^32^,^33^. However, these approaches have been hampered by difficulties in depletion of hypusinated eIF5A, as it is a stable protein with a long half-life. We have employed adenoviral eIF5A shRNA to deplete eIF5A extensively in HeLa cells. Using this system, we have undertaken an unbiased, high-throughput protein expression analyses by iTRAQ (isobaric tags for relative and absolute quantitation) to gain insights into the cellular proteome changes and to identify pathways altered by a deficiency of eIF5A in HeLa cells.

## Results

### Effects of adenoviral eIF5A shRNA transduction on eIF5A level, cell growth, viability and protein synthesis in HeLa cells

**Figure 1:**
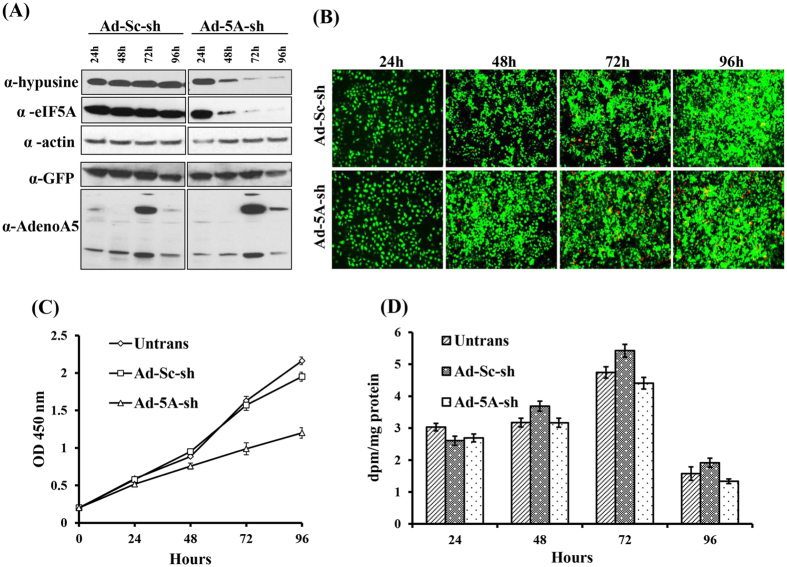
The effects of Ad-eIF5A-shRNA *vs* Ad-scramble-shRNA transduction in HeLa cells. (**A**) eIF5A levels were determined by western blotting using eIF5A antibody (BD Biosciences) and hypusine-specific antibody^34^. GFP and Adeno type 5 antibodies were used to compare the viral load. Actin was used as sample loading control. (**B**) Live cells exhibit green fluorescence due to GFP expressed from both of the adenoviral shRNAs, whereas the dead/dying cells are detected by red fluorescence using LIVE/DEAD cell imaging kit (Dojindo Laboratories). Representative images of three independent experiments are shown. (**C**) Cell proliferation was measured at OD_450_ using Cell Counting Kit-8 assay (Dojindo Laboratories). Representative data was plotted from three independent experiments done in triplicate ± SD. (**D**) The overall rate of cellular protein synthesis was measured by quantitation of [^3^H]leucine incorporation.

To assess the role of eIF5A in HeLa cells, we made a loss-of-function approach, by repressing its expression using adenovirus expressing both eIF5A shRNA and a GFP reporter and comparing the effects eIF5A shRNA with those of scramble shRNA. The eIF5A level was monitored using two different antibodies, one that is specific for hypusine-modified eIF5A^34^ and another that recognizes the non-hypusinated forms of eIF5A as well as the hypusine-modified form. In the first 24 h of eIF5A shRNA transduction, there was only a small decline in eIF5A level, compared to the control (Fig. 1A). At later time points, a remarkable reduction in eIF5A was observed with Ad-eIF5A-shRNA transduction (down to 25–30% at 48 h and to less than 10% after 72 h) while no/little decrease of eIF5A was observed in cells transduced with the control scramble shRNA. The viral titers of Ad-eIF5A-shRNA and Ad-scramble-shRNA were kept the same in this and other experiments, as indicated by the similar levels of GFP fluorescence (Fig. 1B) and as confirmed by western blotting with GFP antibody and Adeno type 5 antibody (Fig. 1A). GFP expression was detected in all cells, indicating close to 100% transduction efficiency.

The live/dead cell imaging (Fig. 1B) displayed increased cell death (red color) after 72 h of Ad-eIF5A-shRNA transduction. The cellular viability and growth patterns were examined by a quantitative colorimetric assay using the Cell Counting Kit-8 (Fig. 1C). HeLa cells treated with scramble shRNA displayed a growth curve similar to that of the untransduced cells up to 72 h. HeLa cells transduced with Ad-eIF5A-shRNA showed a similar growth curve as those of untransduced or scramble shRNA-transduced cells for the first 24 h, but a pronounced growth inhibition was observed after 72 h, concomitant with the reduction of eIF5A below 10% of the normal level. When total protein synthesis was measured by pulse labeling with [^3^H]leucine, the degree of inhibition was relatively small (<20% and <30% at 72 and 96 h, respectively) (Fig. 1D), suggesting that there is no global inhibition of protein synthesis upon depletion of eIF5A.

### iTRAQ identification of proteins whose levels are significantly altered upon depletion of eIF5A

**Figure 2:**
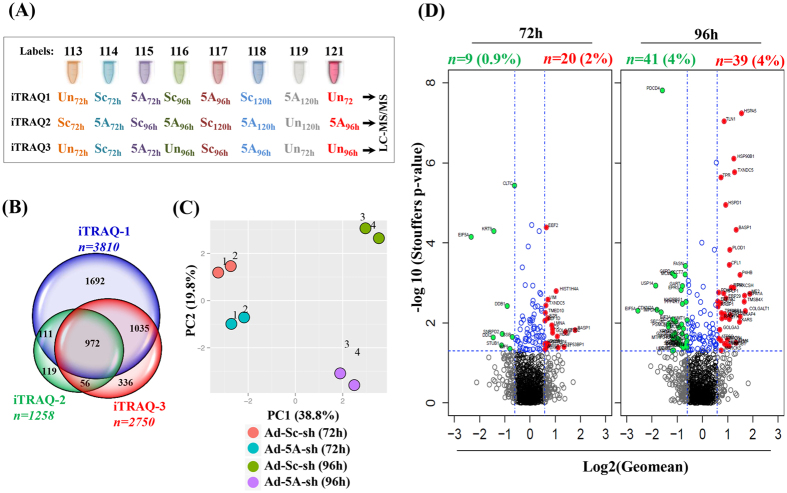
Schematic presentations of three iTRAQ analyses. (**A**) Three independent experiments are indicated by iTRAQ 1, 2 and 3 and the samples labeled with 8 different isobaric tags, 113–119 and 121, are indicated by different colors. (**B**) Venn diagram depicting the number of proteins ‘*n’* identified at all the time points in each iTRAQ and the 972 proteins identified in all three experiments. (**C**) PCA analyses of the samples of iTRAQ3. Each of the 72 h samples (Ad-Sc-sh(72 h), Ad5A-sh(72 h)) was compared against duplicate untransduced samples, Un72h labeled with isobaric tag 113, and Un72h labeled with tag 119, as indicated by 1 and 2, respectively. Each of the 96 h samples (Ad-Sc-sh(96 h), Ad5A-sh(96 h)) was compared against Un96 labeled with 116 and Un96 labeled with 121 as indicated by 3 and 4, respectively. (**D**) The complete set of 972 proteins commonly identified in three experiments is shown as volcano plots. Each data point indicates the protein expression level (log2 value of geometric mean) (X axis) with their corresponding −log10 of Stouffer’s p-value (Y axis). The threshold for differential expression (cut-off = 1.5 fold and significance level of p-value ≤ 0.05) is indicated by dashed blue lines. The significantly decreased and increased proteins are depicted by solid green and red circles, respectively.

We compared the complete proteomes of HeLa cells transduced with Ad-eIF5A-shRNA with those of cells transduced with Ad-scramble-shRNA by the iTRAQ method. After tryptic digestion of total cellular proteins, 8-plex-iTRAQ was performed by labeling separate digested samples individually with one of the eight isobaric tags (Fig. 2A) and the relative levels of each peptide in the mixture of all the labeled samples were estimated by mass spectroscopy after chromatographic separation. The experiments were repeated with three sets of biological replicates (iTRAQ 1, 2 and 3). The changes in total proteome associated with depletion of eIF5A were determined by comparing the protein expression ratios (Ad-eIF5A-sh RNA sample/Ad-scramble-shRNA sample) at 72 and 96 h in each replicate experiment. The unique proteins (with ≥2 unique peptides with >95% confidence interval) were identified from the three experiments, 3810, 1258 and 2750 proteins in iTRAQ1, iTRAQ2 and iTRAQ3, respectively (Fig. 2B), and 972 proteins were identified as common to all three runs. PCA (Principal Component Analysis) based on the relative protein expression levels revealed the existing differences among the four samples of iTRAQ3 (Ad-scramble-shRNA- and Ad-eIF5A-shRNA-transduced at the two time points 72 h and 96 h) (Fig. 2C). Analysis of each of the four samples against two untransduced duplicates generated two proximal circles, indicative of a high similarity of duplicate data and the reliability of the iTRAQ-based quantitation.

The expression datasets of the 972 commonly identified proteins were compiled to generate a final list of detailed protein IDs, gene symbols and relative protein ratios can be found as Supplementary Table S1. A volcano plot of the geometric mean of expression ratios and the combined Stouffer’s p-values of the 972 identified proteins (each protein indicated as a circle) displays a number of cellular proteins whose expression levels were altered upon depletion of eIF5A (Fig. 2D). The cut off values for significant changes in protein ratio, ≥1.5 or ≤0.66 (Log2 values, ≥0.585 or ≤−0.585) and that for p-value, 0.05 (−Log 10, 1.301) are indicated by broken blue lines. At 72 h, in the Ad-eIF5A-shRNA-transduced cell samples, only 0.9% (9, solid green circles) and 2.0% (20, solid red circles) of the 972 proteins were significantly decreased or increased, respectively. At 96 h of transduction, a higher percent of proteins showed significantly altered levels, with 41 decreased proteins (~4%) and 39 increased proteins (~4%). The 104 unique proteins with a significantly altered expression pattern at 72 h and/or 96 h of Ad-eIF5A-shRNA transduction (Table 1) were used for further analyses of polyproline motif and functional ontology.

### Effect of eIF5A shRNA transduction on the levels of polyproline-containing proteins

As a recent study provided evidence for the role of eIF5A in the translation elongation of polyproline motifs in *S. cerevisiae*^21^, we investigated the effect of eIF5A depletion on polyproline-containing proteins of the HeLa cell proteome. 188 of the 972 proteins contained at least 1 PPP motif, but the majority of them did not show considerable difference in levels in Ad-eIF5A-shRNA-transduced cells *vs* Ad-scramble-shRNA-transduced cells at 72 and 96 h of the viral transduction (Table S1). Of the 104 proteins with significantly altered levels (Table 1), 20 proteins contain polyproline motifs (≥1 PPP units) and are listed in the order of the number of PPP units (Table 2). At 72 h of Ad-eIF5A-shRNA transduction, only one (EIF5) out of the 9 decreased proteins and 4 (SF3B2, EZR, GOLGA3, TP53BP1) out of the 20 increased proteins (fulfilling the ratio requirements of ≥1.5 or ≤0.666 with p-values < 0.05) contained polyproline motifs. At 96 h, 9 (KHDRBS1, EIF5, EIF3A, FASN, IMPDH1, NPEPPS, RBM14, SEC24C, U2AF2) out of 41 decreased proteins and 9 (ZYX, BAG3, CKAP4, EEF1B2, GOLGA3, HSPA5, KARS, PRKCSH, TP53BP1) out of 39 increased proteins contained polyproline motifs (Table 1). Among these polyproline proteins identified by iTRAQ, the percent of increased proteins was higher than that of decreased proteins upon eIF5A depletion at both 72 and 96 h. Furthermore, SF3B2 and ZYX, which contain the highest polyprolines (11 and 9 PPP units, respectively) were increased upon eIF5A depletion, whereas KHDRBS1 and EIF5, containing 4 and 3 PPP motifs, respectively, were decreased. As the depletion of eIF5A did not cause consistent reduction of all the polyproline proteins, other factors (in addition to polyproline motifs) must have contributed to changes in these protein levels.

**Figure 3:**
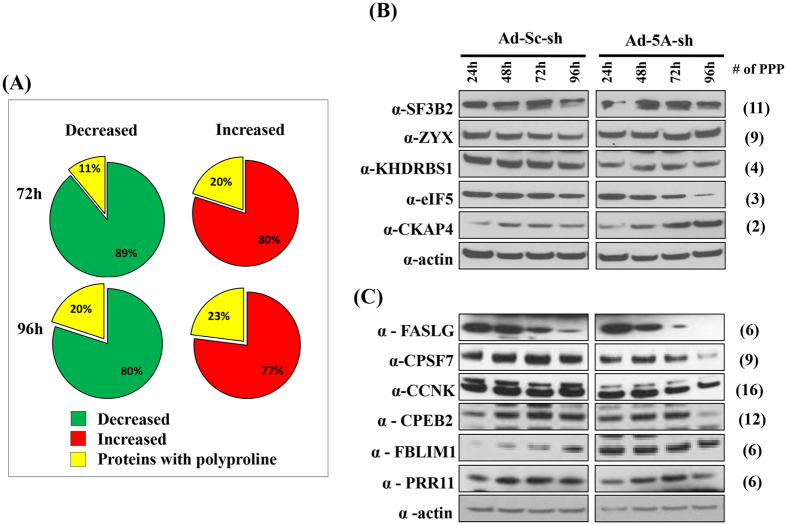
Comparison of levels of polyproline-containing proteins. (**A**) Pie diagrams showing the percent of polyproline proteins in the significantly altered protein pool (Fig. 2C) at 72 and 96 h after Ad-eIF5A-shRNA transduction. The decreased and increased proteins are shown as green and red, respectively, and the polyproline protein fraction is indicated by yellow. (**B**) Western blot validation of altered expression of iTRAQ-identified polyproline proteins. (**C**) Western blot analyses of polyproline proteins not identified by iTRAQ.

We then verified the iTRAQ data of the polyproline proteins by western blotting (Fig. 3B). Three polyprolyl proteins, SF3B2 (splicing factor 3B subunit 2), ZYX (Zyxin, Zn-binding phosphor protein) and CKAP4 (cytoskeleton-associated protein 4) were enhanced in Ad-eIF5A- shRNA-transduced cells, whereas KHDRBS1 and eIF5 levels were reduced (Fig. 3B), consistent with the iTRAQ data. We also checked, by western blotting, the levels of several polyproline-rich proteins that were not identified by iTRAQ (Fig. 3C). Of these, the levels of FASLG, CPSF7, and CPEB2 proteins were reduced in the eIF5A-depleted cells, with a steep decline at 96 h of Ad-eIF5A-shRNA transduction, while CCNK and PRR11 showed moderate decline at 96 h. In contrast, the FBLIM1 protein level remained much higher in Ad-eIF5A-shRNA-transduced cells compared to the Ad-scramble-shRNA-transduced cells at all the time points.

### Functional ontology classification and bioinformatics analyses of differentially regulated proteins

**Figure 4:**
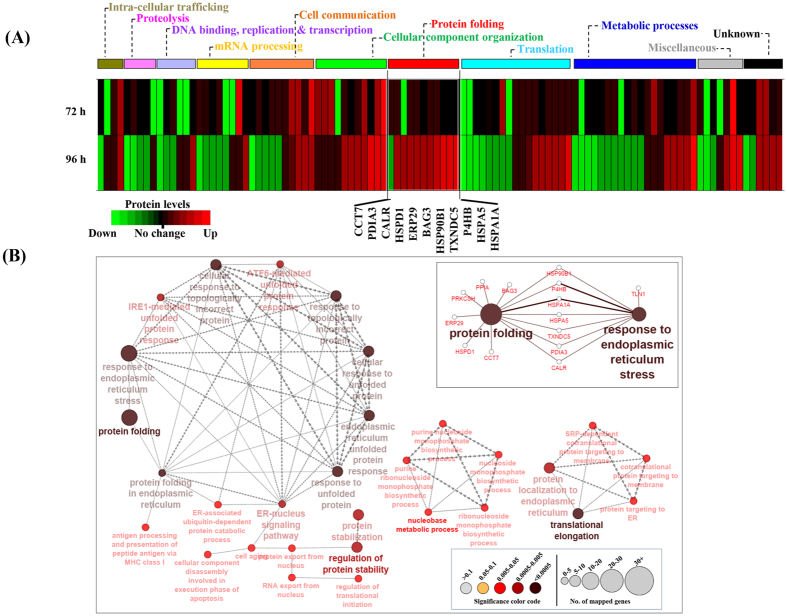
Functional ontology analyses of proteins with significantly altered levels upon depletion of eIF5A. (**A**) The heat map of 104 differentially expressed proteins identified from iTRAQ analyses at two time points (72 and 96 h of adenoviral transduction). The decreased and increased proteins are indicated by range of green and red intensities, respectively. Different functional categories of proteins are indicated with horizontal bars on top. The 11 proteins belonging to the ‘protein folding’ category are indicated under the heat map. (**B**) Three major functional networks obtained from the 104 proteins using Cytoscape software. Each node (filled circle) represents a biological process and the size and color code indicate, respectively, the number of genes and significance of the terms (bottom inset). The direction of network is shown by arrow-head of edges and the edge-thickness is based on kappa-score level calculated automatically by ClueGO. The molecular interaction network between protein folding and response to ER stress is shown in the inset.

In an effort to gain insights into the cellular functions of eIF5A, functional analyses of the significantly altered proteins were performed. Using PANTHER classification system we have integrated the associated molecular functions and biological processes of the 104 proteins whose levels were significantly altered in Ad-eIF5A-shRNA-transduced cells (Table 1). The heatmap in Fig. 4A shows the overrepresentation of significantly altered proteins in several biological processes, including intracellular vesicular trafficking, proteolysis, DNA binding/replication/transcription, mRNA processing, cell communication/signaling, cellular component organization, protein folding, translation, and metabolic processes. Of these, proteins belonging to the ‘Cellular component organization’ and the ‘Protein folding’ categories were prominently increased, whereas the majority of proteins involved in the metabolic processes were decreased. The ‘Protein folding’ category stands out with 10 of 11 proteins (PDIA3, CALR, HSPD1, ERP29, BAG3, HSP90B1, TXNDC5, P4HB, HSPA5, HSPA1A, listed in Table 2) were increased in Ad-eIF5A-shRNA-transduced cells as compared to the Ad-scramble-shRNA-transduced counterpart at 96 h. All of the proteins except BAG3 displayed similarly high or higher fold changes at 120 h (Table S1), indicating intensification of stress conditions with extended deprivation of eIF5A. Increased expression of these proteins and certain other proteins in the ‘Translation’ category may reflect compensatory mechanisms of these cells to cope with eIF5A-deficient conditions.

Further, we explored the functional themes of the above 104 proteins using Cytoscape, a bioinformatics software for visualizing functional and molecular interaction networks. The functional interaction network in Fig. 4B suggests that the depletion of eIF5A in mammalian cells significantly affects the protein folding machinery. The 11 proteins belonging to this ontology term form the biggest and most significant node (p-value > 0.0005) amongst all networks. In the largest network, the ‘response to ER stress’ is the most popular node with the highest number of edges interconnected to 8 different nodes (Fig. 4B) and with 11 different chaperone proteins *i.e.* CCT7, BAG3, HSPD1, ERP29, HSP90B1, P4HB, HSPA1A, HSPA5, TXNDC5, PDIA3, CALR. Therefore, in eIF5A-depleted cells, the protein folding machinery appears to be markedly activated, probably due to ER stress.

### Verification of ER stress and unfolded protein response in eIF5A-depleted HeLa cells by western blotting

**Figure 5:**
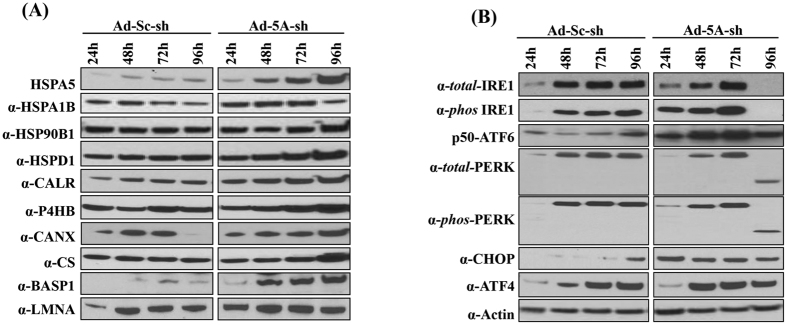
Up-regulation of chaperones and the UPR pathway upon depletion of eIF5A. (**A**) Validation of increased proteins (identified from iTRAQ) including those belonging to ‘Protein folding’ category (Fig. 4A) by western blot. (**B**) The levels of selected proteins involved in triggering ER stress and UPR were measured by western blotting using specific antibodies.

We further validated the increased expression of several iTRAQ-identified proteins by western blotting (Fig. 5A). The typical ER stress marker protein, the 78 kDa glucose-regulated protein precursor (HSPA5/GRP78/BiP), and other chaperones, including heat shock protein 1A/1B (HSPA1B), endoplasmin (HSP90B1), heat shock protein (HSPD1), calreticulin (CALR) and prolyl 4-hydroxylase (P4HB) were markedly increased in Ad-eIF5A-shRNA-transduced cells during 48–96 h, although the time course and the extent of changes varied with different proteins. In addition, an increase in another molecular chaperon, calnexin (CANX) that was detected in iTRAQ (Table S1) was also confirmed by western blotting. Other iTRAQ-identified proteins not belonging to this category, *e.g.* CS (citrate synthase), BASP1 (brain acid soluble protein 1) and LMNA (lamin isoform A) were also confirmed to be increased in Ad-eIF5A-shRNA-transduced cells, compared to Ad-scramble-shRNA-transduced cells (Fig. 5A).

We then examined the levels of the ER stress marker proteins involved in the three canonical pathways of UPR, including inositol-requiring protein 1α (IRE1α), *phos*-IRE1, activating transcription factor 6 (ATF6), protein kinase-like endoplasmic reticulum kinase (PERK) and *phos*-PERK (Fig. 5B). As these proteins were not detected by iTRAQ, their levels were examined by western blotting using specific antibodies. The levels of total-PERK and *phos*-PERK did not exhibit a noticeable increase in eIF5A-depleted cells. Nonetheless, their downstream signaling molecules, ATF4 and CHOP were clearly enhanced between 24–48 h and 24–72 h, respectively, in Ad-eIF5A-shRNA-transduced cells. The *phos*-IRE1 was increased between 24 h to 72 h in the eIF5A shRNA-transduced cells compared to the controls. Most of all, the activated p50-ATF6 level was remarkably enhanced in eIF5A-depleted cells between 24 h to 96 h of the viral transduction, indicative of a major ATF6-mediated UPR with partial contribution of PERK- and IRE1-pathways. The activation of the ATF6 pathway probably led to transcriptional activation of expression of those chaperones listed in Fig. 4A and Table 2. Curiously, we observed consistent disappearance of IRE1, *phos*-IRE1, PERK, *phos*-PERK and several other proteins at 96 h of AD-eIF5A-shRNA transduction. In the cases of PERK and *phos*-PERK, a protein band at 50–55 kDa was detected instead of ~125 kDa at 96 h, suggesting a specific proteolytic cleavage of PERK between 72–96 h of Ad-eIF5A-shRNA transduction. Since eIF5A was extensively depleted by 72 h of Ad-eIF5A-shRNA transduction (Fig. 1A), a sudden decline of these proteins at 96 h might be secondary effect possibly due to other changes, *e.g.* activation of cellular proteolytic machinery or denaturation of these proteins upon prolonged exposure to eIF5A-deficient cellular environment, rather than a direct consequence of eIF5A depletion on their synthesis.

## Discussion

Eukaryotic initiation factor 5A (eIF5A) is an essential cellular factor with a unique amino acid, hypusine, formed post-translationally only in this protein. This putative translation factor has been implicated in a variety of cellular processes including mRNA decay^22^ cell cycle progression^26^, apoptosis^29^, cell polarity^23^,^24^, retroviral infection^26^,^28^, translation elongation at polyproline sites^21^ and stress responses^35^. In spite of decades of research, the true physiological function of this protein and its critical hypusine residue has remained enigmatic^2^,^3^,^4^. At the molecular level, a recent report suggested that, like its bacterial ortholog EF-P, eIF5A can alleviate ribosome stalling at polyproline stretches and promote synthesis of polyproline-rich proteins in *S. cerevisiae*^21^. However, there has not been definitive experimental evidence that could substantiate this conjecture either by global proteomic analyses or ribosome profiling of eIF5A-deficient cells. In the current study, we have made an iTRAQ approach to assess the outcome of loss of eIF5A on the total proteome of HeLa cells and to identify the cellular pathways and processes affected by eIF5A depletion. Our proteomic data reveal alterations in several specific cellular pathways, with particularly strong increase in the level of a number of chaperone proteins in Ad-eIF5A-shRNA-transduced cells and suggest ER stress and primarily ATF6-mediated UPR in eIF5A-deficient cells.

Our data show that HeLa cells depleted of eIF5A undergo growth arrest and eventual apoptosis. However, the growth inhibition cannot be solely attributed to an overall inhibition of protein synthesis, because there was no pronounced inhibition of total protein synthesis in Ad-eIF5A-shRNA-transduced HeLa cells compared to their Ad-scramble-shRNA-transduced counterparts. This is in agreement with the hypothesis that eIF5A affects the translation of only a subset of mRNAs and is consistent with the relatively small effects of eIF5A depletion on the overall protein synthesis rate and a small portion of proteins (104 out of the 972 proteins commonly identified in three iTRAQ experiments) significantly altered in levels at 72 and/or 96 h of Ad-eIF5A-shRNA transduction.

The role of EF-P in alleviating ribosome stalling at polyproline stretches is well established, although target of EF-P may not be limited to polyproline stretches^15^ and other factors *i.e.*, the upstream sequence context^36^ and translation initiation rate^37^ may affect ribosome stalling in bacteria. In spite of the general assumption for the same mechanism of action for EF-P and eIF5A, there has been only one major report on the effect of eIF5A on polyproline elongation^21^. Our data show that polyproline-containing proteins were either increased or decreased in Ad-eIF5A-shRNA-transduced HeLa cells (Fig. 3) with no exclusive effect of eIF5A depletion on the polyproline-containing protein levels. The mechanistic role of eIF5A on the relief of ribosome stalling and the translation of polyproline motifs has not yet been demonstrated in mammalian cells or cell-free lysates and no ribosome profile data have been reported. Previous proteomic studies employed inhibitors of the hypusine biosynthesis enzymes, DHS or DOHH, siRNA or stable eIF5A shRNA cells for depletion of eIF5A^32^,^33^. None of those studies achieved as drastic depletion of eIF5A as did the Ad-eIF5A-shRNA transduction used in our work. The first proteomic study^32^ identified, by 2D PAGE separation, several cancer related proteins differentially expressed in cervical cancer cells treated with a DOHH inhibitor or eIF5A siRNA. Another study^33^ examined proteomic changes in pancreatic cancer cells stably transfected with eIF5A shRNA or treated with a DHS inhibitor by using one dimensional PAGE and spectral counting method, which does not yield as precise analysis as iTRAQ. While these studies focused on different altered pathways, neither their data nor our current data offer substantial support for a specific or exclusive role for eIF5A in the synthesis and maintenance of the levels of polyproline-containing proteins. Thus, elucidation of the mechanism of eIF5A in eukaryotic translation warrants further investigation.

Ad-eIF5A-shRNA transduction provided the most effective means to deplete eIF5A, with nearly 100% infection of HeLa cells. However, due to the long half-life of eIF5A, it took nearly 72 hours to deplete eIF5A below 10% of the control level and the proteomic changes appeared to be delayed. Inhibition of growth was also delayed, starting around 48 h of eIF5A shRNA treatment, when eIF5A is reduced to 25% of normal level. This finding is consistent with the notion that eIF5A is a stable protein that exists in large excess over the minimum level required for normal growth in mammalian cells. Furthermore, eIF5A is not a general translation factor, but appears to regulate the translation of only a subset of mRNAs^18^,^38^. In view of these features of eIF5A and the long average half-life (20 h) of HeLa cell proteins^39^, it is not surprising that a relatively small number of proteins were significantly altered in level (protein ratio ≥1.5 or ≤0.66, p-value < 0.05) at 72 h. However, proteome changes were markedly enhanced at 96 h of eIF5A shRNA treatment. Although maximum variance in iTRAQ data was observed at 120 h of adenoviral transduction, we did not include this data for further analyses, because of potential secondary effects associated with prolonged depletion of eIF5A. In this regard, it is not possible to segregate the primary effects of eIF5A depletion from indirect secondary effects, as depletion of eIF5A takes a long time and is accompanied by inhibition of cellular growth. Furthermore, the cellular proteome can be altered by changes not only in synthesis rate of individual proteins, but also by changes in their turnover rates.

The ontology classification and functional interaction networks revealed over-representation of proteins (significantly altered in levels upon eIF5A depletion) in the ‘protein folding’ and ‘response to unfolded proteins’ categories, as two major associated biological processes. Accumulation of unfolded or misfolded proteins in the lumen of the ER leads to the activation of UPR that plays a critical role in restoring homeostasis^40^. In case of canonical UPR, the cells respond to ER stress by three pathways: i) halting protein translation, ii) proteasomal degradation of misfolded proteins, and iii) activation of the signaling pathways that lead to increase the production of molecular chaperones involved in protein folding. If the above objectives are not met within a certain time span, cells may undergo apoptosis. In the eIF5A-depleted cells, increased level of the chaperone proteins persisted up to 120 h of Ad-eIF5A-shRNA transduction (data not shown). p50-ATF6 was highly up-regulated at all time points and this probably led to the increased levels of general chaperones such as HSPE1, HSPD1/Hsp60, HSPA8, HSPA1A/Hsp70, and ER specific chaperones like HSPA5/Bip (Grp78), HSP90B1, Calreticulin, Calnexin, HSP90B1(Grp94) that were observed in iTRAQ data and validated by western blotting. We also observed an increase in *phos*-IRE1-α at 24–72 h of Ad-eIF5A-shRNA transduction, without a notable increase in IRE1- α or PERK. In this regard, eIF5A depletion-induced UPR seems to be somewhat different from canonical UPR.

Interestingly, our finding in mammalian cells bears analogy to certain phenotypes observed in *S. cerevisiae* temperature-sensitive eIF5A mutant strains^25^,^41^. Loss of eIF5A function led to impairment of the co-translational translocation of proteins into the ER and up-regulation of stress-induced chaperones, Hsp31, Sse1, Ssb2 and Ssa1^25^. Furthermore, a mutation in YPT1 gene, encoding an essential protein involved in ER-to-Golgi vesicular transport, caused synthetic lethality in an eIF5A mutant strain^41^, suggesting a connection between translation and the secretary pathway in terms of eIF5A function. Regarding the precise molecular mechanisms, it is unknown whether eIF5A deficiency-induced stress response is mediated solely by impaired polyproline synthesis and how the UPR signaling pathways are triggered upon depletion of eIF5A. It is possible that ER stress is caused by the accumulation of incomplete polypeptide fragments (ribosome fall-off products) or of misfolded proteins, or by impaired vesicular transport when eIF5A is limiting. Future studies will be directed to delineate the sequence of molecular events resulting from eIF5A deficiency leading to changes in cellular pathways including those of protein folding and UPR.

## Experimental Procedures

### Methods

#### Cell Culture and Adenoviral Transduction

HeLa cells were cultured in DMEM supplemented with 10% heat-inactivated FBS. For adenoviral transduction, cells were trypsinized and resuspended in DMEM containing 10% FBS at the density of 1 × 10^6^ cells/ml. Ad-GFP-U6-h-EIF5A-shRNA or Ad-GFP-U6-scramble-shRNA (Vector Biolabs) was added to the trypsinized cell suspension at the multiplicity of infection (MOI) of 60. After mixing the cells with virus for 30 min, they were seeded in tissue culture dishes.

#### Measurement of protein synthesis rate: Pulse–labelling with [^3^H]leucine

Cells were radiolabeled with [^3^H]leucine (PerkinElmer Life Sciences), by incubation in leucine-free DMEM containing 10% FBS and 20 μCi/mL of [^3^H]leucine for 30 min. Labelled cells were washed with ice-cold PBS, harvested, and precipitated with 10% TCA. The precipitated proteins were dissolved in 0.1 ml of 0.15N NaOH and the radioactivity was determined using a liquid scintillation counter (Beckman Coulter).

#### Protein extraction and peptide labeling

Cells were lysed in phosphate buffer saline (containing 0.05% SDS, freshly prepared protease inhibitor cocktail (ThermoScientific Co.) and 1mM PMSF) by sonication and the protein concentration in each sample was measured by a bicinchoninic acid assay. From each sample 50 μg of protein was precipitated by stepwise addition of 3 aliquots of cold acetone^42^ while vortexing. The tryptic digestion of the protein samples, labelling, separation of labeled peptides by two dimensional liquid chromatography (2D-LC) followed by mass spectrometry and protein identification were performed at the Proteomics and Mass Spectrometry Core, Research Facility of the College of Medicine, Pennsylvania State University as follows. The lysate proteins were treated with the reducing agent TCEP (tris-(2-carboxyethyl) phosphine) (Pierce, 20490) and alkylating agent iodoacetamide. The trypsin digestion was performed with Sequencing Grade Modified Trypsin, (Promega, V511) with protease: protein ratio of 1:100 overnight at 48°C in 50 mM ammonium bicarbonate buffer. Each digested sample was individually labeled with one of the 8 isobaric tags according to a general protocol in the manufacturer’s manual (Applied Biosystems, iTRAQ Reagent-8Plex Multiplex Kit, 4390812, manual: http://www.absciex.jp/Documents/Downloads/Literature/mass-spectrometry-4375249C.pdf). The labeled samples were combined, dried and resuspended in 500 μl of 12 mM ammonium formate for Strong CationeXchange (SCX).

#### Two dimensional liquid chromatography

SCX separations were performed on a passivated Waters 600E HPLC system, using a 4.6 × 250 mm PolySULFOETHYL Aspartamide column (PolyLC, Columbia, MD) at a flow rate of 1 ml/min. The gradient was Buffer-A (10 mM ammonium formate, pH 2.7, in 20% acetonitrile/80% water) at 100% (0–22 min following sample injection), 0% → 40% Buffer B (666 mM ammonium formate, pH 2.7, in 20% acetonitrile/80% water, 16–48 min), 40% → 100% Buffer B (48–49 min), then isocratic 100% Buffer B (49–56 min), then at 56 min back to 100% A to re-equilibrate for the next injection. First 26 ml of fractions were combined into one fraction and 32 additional 1 ml fractions were collected and dried down and resuspended in 9 μl of 2% (v/v) acetonitrile, 0.1% (v/v) formic acid, for the 2^nd^ dimension separation by reverse phase nanoflow LC. The SCX samples were filtered and auto injected using Eksigent NanoLC-Ultra-2D Plus and Eksigent cHiPLC Nanoflex onto a Trap Column (200 μm × 0.5 mm Chrom XP C18-CL 3 μm 120 Å) and eluted through a Nano cHiPLC Column (75 μm × 15 cm Chrom XP C18-CL 3 μm 120 Å). The elution gradient was 95% C (degassed 0.1% formic acid in water)/5% D (degassed 0.1% formic acid in acetonitrile) (300 nl per minute flow rate) to 65% C/35% D in 120 minutes, 15% C/85% D from 120 to 130 minutes, then back to 95% C/5% D from 130–150 min.

#### Mass Spectrometry analysis

The Eluate from reverse phase nanoflow LC was delivered into the ABSciex 5600 TripleTOF mass spectrometer with a NanoSpray III source and using a 10 mm id nanospray tip (New Objective, Woburn, MA). Typical Mass Spectrometer settings used were curtain gas = 25, Gas1 = 4–6, Gas2 = 0, an ionspray floating voltage 2200, and a rolling collision energy voltage was used for CID (collision induced dissociation) fragmentation for MS/MS spectra acquisitions. Each cycle consisted of a TOF-MS spectrum acquisition for 250 ms (mass range 400–1250 Da), followed by information-dependent acquisition of up to 50 MS/MS spectra (50 ms each) of MS peaks above intensity 150 (TOF mass range 65–1600 Da) with a charge state between 2 and 5, taking 2.8 seconds total per full cycle. Once MS/MS fragment spectra were acquired for a particular mass, the mass was dynamically excluded for 6 seconds. Each sample fraction was analysed following a calibration run using trypsin-digested beta-galactosidase as a calibrant followed by a blank run.

#### Protein identification and relative quantification

The protein identification and quantitation was performed using the Paragon algorithm as implemented in Protein Pilot 5.0 software (ProteinPilot5.0, which contains the Paragon Algorithm 5.0.0.0, build 1654 from ABI/MDS-Sciex). As for the ProteinPilot search parameters, trypsin was selected as the digestion enzyme and iodoacetic acid as the cysteine modification agent. In order to search the database the processing parameters were set to ‘biological modification’ and ‘amino acid substitutions’ as ‘ID focus’ for a ‘thorough ID search effort’. The combined spectra were searched against the species-specific sequence database, NCBInr RefSeq Human database (January 02, 2015) containing 29,886 human protein sequences, concatenated with a reversed ‘decoy’ version of the same database and 536 common lab contaminants (ABSciex_ContaminantDB_20070711). The data sets were normalized to the central tendency of the protein ratios to be unity so that a ratio of 1.0 would represent no relative change in that protein’s level among the samples compared. A data-dependent auto-bias correction was performed for each set of iTRAQ ratios such that the adjusted distribution of iTRAQ ratios observed had a median value of 1.0 (or 0 in log space), thus normalized against any minor discrepancies in total protein amount labeled or efficiency of individual iTRAQ labeling in the different samples. Local FDR (false discovery rate) was estimated based on decoy database false positive IDs using the PSPEP algorithm^43^. Redundant protein IDs were collapsed into a MIAPE-compliant single representative ID using the Progroup algorithm in ProteinPilot, and protein IDs were accepted as correctly identified if they had an estimated local FDR ≤0.05.

#### iTRAQ data analyses and identification of differentially expressed proteins

The raw dataset of proteins labelled with all the 8 iTRAQ reagents was refined by removing duplicate entries, contaminants and by filtering using three independent parameters *i.e.* p-value, the number of peptides and the confidence interval used to identify the proteins. In the three iTRAQ experiments the relative change in protein ratio was considered to be significant if the p-value was ≤0.05, (calculated by the ProteinPilot™ 5.0 software based on the ratios of each identified peptide) and if more than 2 peptides per protein were identified with at least 95% confidence level. This selection resulted in 3810, 1258 and 2750 proteins, respectively, as uniquely identified proteins from the iTRAQ experiments, 1, 2 and 3. In these three pools, 972 proteins commonly detected in all three iTRAQ experiments were identified. For quantitative comparison of biological samples, the relative abundance of proteins was determined as the geometric mean of the protein ratios from three replicate experiments. To identify the differentially expressed proteins, a recently developed alternative Meta-analysis approach (Stouffer’s method) was implemented based on combining p-values across different iTRAQ runs arising from the testing of the same null hypothesis from k independent studies^44^. Combining the *p*-values using Stouffer’s method in fact, incorporates the information about ratio quantification confidence and thus is likely to favor proteins identified with high confidence and does not penalize minor run to run variations^44^. Since the method has a bias toward proteins with a higher identification confidence, we therefore have made sure that for a combined *p*-value calculation the respective protein must have been identified with at least two peptides in all three runs. This method has advantages of high tolerance of run variability, low false discovery rate, and emphasis on proteins identified with high confidence. Proteins with geometric mean of ratios ≤0.66 or ≥1.50 and combined Stouffer’s p-value ≤ 0.05, were considered to be significantly up or down- regulated.

### Statistical Analysis

The statistical analysis for proteomics data was conducted within the R computational environment (http://www.r-project.org/)^45^. Statistical significance was set at p ≤ 0.05.

#### Principal component analysis (PCA)

PCA was performed using R computational environment^45^. In the PCA plot, each point represents the variation of a single sample in a multi-dimensional protein expression space.

#### Bioinformatics Analyses: Gene Ontology Classification

For the functional classification of differentially expressed proteins, an ontology database PANTHER (http://pantherdb.org/) was used^46^. The heatmap was constructed using Partek^®^ Genomics Suite^®^ software, version 6.6 Copyright^©^; 2015, Partek Inc., St. Louis, MO, USA. The network visualisation of the biological functions related to the proteins was performed using Cytoscape software with their plug-ins^47^. In order to create a functional network by selecting ClueGO: function, GO: biological process, all the network evidences and only the terms with various levels of significance (p-value < 0.1–<0.0005) were taken into consideration in plug-in ClueGO^48^, which was followed by enrichment of functional network with plug-in CluePedia^49^. The identification of proteins containing polyproline motifs and computation of PPP units were performed using the Perl (practical extraction and report language, https://www.perl.org/) as described earlier^50^.

#### Validation of differentially expressed proteins by western blotting

Cellular proteins were extracted by sonication in PBS containing 0.05% SDS, freshly prepared protease inhibitor cocktail and 1mM PMSF and 25 μg of proteins were used for western blotting. Selected up- or down-regulated proteins detected from iTRAQ analyses were validated by western blotting using β-actin as a loading control.

## Additional Information

**How to cite this article**: Mandal, A. *et al.* Global quantitative proteomics reveal up-regulation of endoplasmic reticulum stress response proteins upon depletion of eIF5A in HeLa cells. *Sci. Rep.* **6**, 25795; doi: 10.1038/srep25795 (2016).

## Supplementary Material

Supplementary Information

## Figures and Tables

**Table 1:** List of proteins with a significantly altered expression pattern.

#	Gene Symbol	Gene details	# of PPP-motifs	GO terms	Protein ratio (72 h)	Protein ratio (96 h)
1	SEC24C	protein transport protein Sec24C	1	Intra-cellular trafficking	0.74	0.39*
2	CLTC	clathrin heavy chain 1 isoform 1	0	Intra-cellular trafficking	0.65*	0.88
3	TMED10	transmembrane emp24 domain-containing protein 10 precursor	0	Intra-cellular trafficking	1.52*	1.03
4	GOLGA3	golgin subfamily A member 3 isoform 1	1	Intra-cellular trafficking	1.86*	1.61*
5	USP14	ubiquitin carboxyl-terminal hydrolase 14 isoform a	0	Proteolysis	0.81	0.28*
6	PSMC4	26S protease regulatory subunit 6B isoform 1	0	Proteolysis	1.09	0.39*
7	NPEPPS	puromycin-sensitive aminopeptidase	1	Proteolysis	0.95	0.5*
8	LONP1	lon protease homolog, mitochondrial isoform 1 precursor	0	Proteolysis	0.91	0.52*
9	STUB1	E3 ubiquitin-protein ligase CHIP isoform a	0	Proteolysis	0.46*	0.68
10	MCM2	DNA replication licensing factor MCM2	0	DNA binding, replication & transcription	0.63	0.47*
11	GTF2I	general transcription factor II-I isoform 5	0	DNA binding, replication & transcription	0.76	0.54*
12	MCM5	DNA replication licensing factor MCM5	0	DNA binding, replication & transcription	1.53	0.59*
13	DDB1	DNA damage-binding protein 1	0	DNA binding, replication & transcription	0.53*	0.89
14	DDI2	protein DDI1 homolog 2	0	DNA binding, replication & transcription	0.36*	1.39
15	TCEB2	transcription elongation factor B polypeptide 2 isoform a	0	DNA binding, replication & transcription	0.69	2.15*
16	U2AF2	splicing factor U2AF 65 kDa subunit isoform b	1	mRNA processing	1.24	0.45*
17	NOB1	RNA-binding protein NOB1	0	mRNA processing	1.04	0.51*
18	HTATSF1	HIV Tat-specific factor 1	0	mRNA processing	1.00	0.57*
19	KHDRBS1	KH domain-containing, RNA-binding, signal transduction-associated protein 1 isoform 1	4	mRNA processing	1.39*	0.64*
20	SF3B3	splicing factor 3B subunit 3	0	mRNA processing	0.62	0.65*
21	SNRPD2	small nuclear ribonucleoprotein Sm D2 isoform 1	0	mRNA processing	0.47*	0.87
22	SF3B2	splicing factor 3B subunit 2	11	mRNA processing	2.65*	1.03
23	TPR	nucleoprotein TPR	0	mRNA processing	0.83	1.67*
24	CDKN2A	cyclin-dependent kinase inhibitor 2A isoform p16INK4a	0	Cell communication	0.95	0.29*
25	SLK	STE20-like serine/threonine-protein kinase isoform 1	0	Cell communication	1.31*	0.51*
26	IQGAP1	ras GTPase-activating-like protein IQGAP1	0	Cell communication	0.76*	0.59*
27	NAMPT	nicotinamide phosphoribosyltransferase precursor	0	Cell communication	1.15	0.64*
28	RBM14	RNA-binding protein 14 isoform 1	1	Cell communication	1.23	0.65*
29	OXSR1	serine/threonine-protein kinase OSR1	0	Cell communication	1.58*	0.94
30	LMNA	lamin isoform A	0	Cell communication	1.82*	1.44*
31	HDGF	hepatoma-derived growth factor isoform a	0	Cell communication	2.12*	1.54
32	MARCKS	myristoylated alanine-rich C-kinase substrate	0	Cell communication	1.05	1.67*
33	TP53BP1	tumor suppressor p53-binding protein 1 isoform 3	1	Cell communication	2.54*	1.69*
34	EZR	ezrin	2	Cellular component organization	1.63*	0.80
35	VIM	vimentin	0	Cellular component organization	1.63*	1.21*
36	HIST1H4A	histone H4	0	Cellular component organization	2.05*	1.31
37	KRT9	keratin, type I cytoskeletal 9	0	Cellular component organization	0.37*	1.37*
38	TLN1	talin-1	0	Cellular component organization	1.18*	1.82*
39	HIST1H1C	histone H1.2	0	Cellular component organization	1.00	1.84*
40	CFL1	cofilin-1	0	Cellular component organization	1.38	2.1*
41	ZYX	zyxin	9	Cellular component organization	1.72	2.1*
42	TPM4	tropomyosin alpha-4 chain isoform Tpm4.2cy	0	Cellular component organization	0.93	2.52*
43	CKAP4	cytoskeleton-associated protein 4	2	Cellular component organization	1.67	2.82*
44	TMSB4X	thymosin beta-4	0	Cellular component organization	3.27	3.16*
45	CCT7	T-complex protein 1 subunit eta isoform a	0	Protein folding	1.05	0.62*
46	PDIA3	protein disulfide-isomerase A3 precursor	0	Protein folding	1.07	1.56*
47	CALR	calreticulin precursor	0	Protein folding	0.65	1.64*
48	HSPD1	60 kDa heat shock protein, mitochondrial	0	Protein folding	1.13	1.9*
49	ERP29	endoplasmic reticulum resident protein 29 isoform 1 precursor	0	Protein folding	1.33	1.91*
50	BAG3	BAG family molecular chaperone regulator 3	2	Protein folding	0.83	1.99*
51	HSP90B1	endoplasmin precursor	0	Protein folding	1.24*	2.37*
52	TXNDC5	thioredoxin domain-containing protein 5 isoform 1 precursor	0	Protein folding	1.55*	2.42*
53	P4HB	protein disulfide-isomerase precursor	0	Protein folding	0.88*	2.81*
54	HSPA5	78 kDa glucose-regulated protein precursor	1	Protein folding	0.91	2.93*
55	HSPA1A	heat shock 70 kDa protein 1A/1B	0	Protein folding	0.76	3.18*
56	eIF5A	eukaryotic translation initiation factor 5A	0	Translation	0.20*	0.17*
57	EIF5	eukaryotic translation initiation factor 5	3	Translation	0.58*	0.42
58	EIF3A	eukaryotic translation initiation factor 3 subunit A	1	Translation	0.87	0.44*
59	DDX21	nucleolar RNA helicase 2 isoform 1	0	Translation	0.78*	0.48*
60	EPRS	bifunctional glutamate/proline–tRNA ligase	0	Translation	1.02	0.56*
61	QARS	glutamine–tRNA ligase isoform a	0	Translation	0.91	0.56*
62	GTPBP4	nucleolar GTP-binding protein 1	0	Translation	1.64*	0.58
63	RPS3	40S ribosomal protein S3 isoform 1	0	Translation	0.56	0.64*
64	EEF2	elongation factor 2	0	Translation	1.57*	0.79*
65	RPS18	40S ribosomal protein S18	0	Translation	1.51*	1.41
66	EEF1D	elongation factor 1-delta isoform 1	0	Translation	1.51*	1.44
67	EEF1B2	elongation factor 1-beta	1	Translation	1.15	1.52*
68	RRBP1	ribosome-binding protein 1	0	Translation	0.98	1.54*
69	RPL4	60S ribosomal protein L4	0	Translation	1.19	1.71*
70	RPLP1	60S acidic ribosomal protein P1 isoform 1	0	Translation	1.16*	1.79*
71	RPN1	dolichyl-diphosphooligosaccharide–protein glycosyltransferase subunit 1 precursor	0	Translation	1.29*	2.21*
72	KARS	lysine–tRNA ligase isoform 2	1	Translation	1.45	2.77*
73	IMPDH1	inosine-5′-monophosphate dehydrogenase 1 isoform a	1	Metabolic processes	1.24	0.32*
74	GSTM3	glutathione S-transferase Mu 3	0	Metabolic processes	0.50	0.44*
75	G6PD	glucose-6-phosphate 1-dehydrogenase isoform a	0	Metabolic processes	0.81	0.44*
76	MTHFD1L	monofunctional C1-tetrahydrofolate synthase, mitochondrial isoform 2 precursor	0	Metabolic processes	0.85	0.45*
77	RRM1	ribonucleoside-diphosphate reductase large subunit	0	Metabolic processes	0.95	0.53*
78	SLC3A2	4F2 cell-surface antigen heavy chain isoform c	0	Metabolic processes	1.24	0.56*
79	HADHA	trifunctional enzyme subunit alpha, mitochondrial precursor	0	Metabolic processes	1.19*	0.57*
80	PPP4R1	serine/threonine-protein phosphatase 4 regulatory subunit 1 isoform b	0	Metabolic processes	0.63	0.58*
81	GART	trifunctional purine biosynthetic protein adenosine-3 isoform 1	0	Metabolic processes	0.84	0.58*
82	FASN	fatty acid synthase	1	Metabolic processes	1.07*	0.63*
83	NMT1	glycylpeptide N-tetradecanoyltransferase 1	0	Metabolic processes	1*	0.66*
84	PDXK	pyridoxal kinase	0	Metabolic processes	1.5*	1.08
85	CS	citrate synthase, mitochondrial precursor	0	Metabolic processes	1.86*	1.18
86	MPST	3-mercaptopyruvate sulfurtransferase isoform 2	0	Metabolic processes	1.56*	1.23
87	ATP5O	ATP synthase subunit O, mitochondrial precursor	0	Metabolic processes	0.88	1.57*
88	ATP5A1	ATP synthase subunit alpha, mitochondrial isoform a precursor	0	Metabolic processes	0.88*	1.84*
89	MDH2	malate dehydrogenase, mitochondrial isoform 1 precursor	0	Metabolic processes	1.40	1.89*
90	PRKCSH	glucosidase 2 subunit beta isoform 2 precursor	1	Metabolic processes	1.46	2.37*
91	ME2	NAD-dependent malic enzyme, mitochondrial isoform 1 precursor	0	Metabolic processes	1.81	3.68*
92	UBE2M	NEDD8-conjugating enzyme Ubc12	0	Miscellaneous	1.54	0.44*
93	KPNA2	importin subunit alpha-1	0	Miscellaneous	0.59	0.47*
94	PPIA	peptidyl-prolyl cis-trans isomerase A isoform 1	0	Miscellaneous	0.95	0.65*
95	SSB	lupus La protein	0	Miscellaneous	0.62*	0.71
96	PLOD1	procollagen-lysine, 2-oxoglutarate 5-dioxygenase 1 precursor	0	Miscellaneous	1.07	2.12*
97	BASP1	brain acid soluble protein 1	0	Miscellaneous	3.45*	2.53*
98	COLGALT1	procollagen galactosyltransferase 1 precursor	0	Miscellaneous	0.94	3.25*
99	PDCD4	programmed cell death protein 4 isoform 3	0	Unknown	0.97*	0.33*
100	TRMT112	multifunctional methyltransferase subunit TRM112-like protein isoform 1	0	Unknown	0.67	0.63*
101	RCN1	reticulocalbin-1 precursor	0	Unknown	2.17*	1.58
102	GPATCH4	G patch domain-containing protein 4 isoform 1	0	Unknown	1.66	1.66*
103	NENF	neudesin precursor	0	Unknown	1.03	1.95*
104	HMGB1	high mobility group protein B1	0	Unknown	1.12	2.08*

The proteins with ratios of ≥1.5 or ≤0.666 (eIF5A shRNA- transduced sample *vs* scramble shRNA-transduced counterparts) and with a combined Stouffer’s p-value ≤ 0.05 at either 72 or 96 h point were selected. The ratios are given as geometric means from three independent iTRAQ experiments and the ratios with combined Stouffer’s p-value ≤ 0.05 are indicated by asterisk (*).

**Table 2:** List of polyproline-containing proteins commonly identified from three independent iTRAQ experiments.

#	Gene Symbol	Gene details	Protein ratio (72 h)	Protein ratio (96 h)	# of PPP-motifs
1	SF3B2	splicing factor 3B subunit 2	2.65*	1.03	11
2	ZYX	zyxin	1.72	2.1*	9
3	KHDRBS1	KH domain-containing, RNA-binding, signal transduction-associated protein 1 isoform 1	1.39*	0.64*	4
4	EIF5	eukaryotic translation initiation factor 5	0.58*	0.42	3
5	BAG3	BAG family molecular chaperone regulator 3	0.83	1.99*	2
6	CKAP4	cytoskeleton-associated protein 4	1.67	2.82*	2
7	EZR	ezrin	1.63*	0.80	2
8	EEF1B2	elongation factor 1-beta	1.15	1.52*	1
9	EIF3A	eukaryotic translation initiation factor 3 subunit A	0.87	0.44*	1
10	FASN	fatty acid synthase	1.07*	0.63*	1
11	GOLGA3	golgin subfamily A member 3 isoform 1	1.86*	1.61*	1
12	HSPA5	78 kDa glucose-regulated protein precursor	0.91	2.93*	1
13	IMPDH1	inosine-5′-monophosphate dehydrogenase 1 isoform a	1.24	0.32*	1
14	KARS	lysine–tRNA ligase isoform 2	1.45	2.77*	1
15	NPEPPS	puromycin-sensitive aminopeptidase	0.95	0.5*	1
16	PRKCSH	glucosidase 2 subunit beta isoform 2 precursor	1.46	2.37*	1
17	RBM14	RNA-binding protein 14 isoform 1	1.23	0.65*	1
18	SEC24C	protein transport protein Sec24C	0.74	0.39*	1
19	TP53BP1	tumor suppressor p53-binding protein 1 isoform 3	2.54*	1.69*	1
20	U2AF2	splicing factor U2AF 65 kDa subunit isoform b	1.24	0.45*	1

The ratios of each protein in Ad-eIF5A-shRNA-transduced cells *vs* Ad-scramble-shRNA-transduced cells are shown at 72 and 96 h of transduction. The geometric mean of ratios from the three iTRAQ data sets is given at each time point and those ratios with p-value ≤ 0.05 are indicated by an asterisk (*) superscript. The computed number of polyproline motifs (PPP) is given for each protein.

## References

[b1] Kemper W. M., Berry K. W. & Merrick W. C. Purification and properties of rabbit reticulocyte protein synthesis initiation factors M2Balpha and M2Bbeta. J. Biol. Chem. 251, 5551–5557 (1976). 965377

[b2] Rossi D., Kuroshu R., Zanelli C. F. & Valentini S. R. eIF5A and EF-P: two unique translation factors are now traveling the same road. Wiley interdisciplinary reviews. RNA 5, 209–222, 10.1002/wrna.1211 (2014). 24402910

[b3] Dever T. E., Gutierrez E. & Shin B. S. The hypusine-containing translation factor eIF5A. Crit. Rev. Biochem. Mol. Biol. 49, 413–425, 10.3109/10409238.2014.939608 (2014). 25029904 PMC4183722

[b4] Mathews M. B. & Hershey J. W. The translation factor eIF5A and human cancer. Biochim. Biophys. Acta 1849, 836–844, 10.1016/j.bbagrm.2015.05.002 (2015). 25979826 PMC4732523

[b5] Park M. H., Cooper H. L. & Folk J. E. Identification of hypusine, an unusual amino acid, in a protein from human lymphocytes and of spermidine as its biosynthetic precursor. Proc. Natl. Acad. Sci. USA 78, 2869–2873 (1981). 6789324 10.1073/pnas.78.5.2869 PMC319460

[b6] Cooper H. L., Park M. H., Folk J. E., Safer B. & Braverman R. Identification of the hypusine-containing protein hy+ as translation initiation factor eIF-4D. Proc. Natl. Acad. Sci. USA 80, 1854–1857 (1983). 6403941 10.1073/pnas.80.7.1854 PMC393708

[b7] Park M. H. The post-translational synthesis of a polyamine-derived amino acid, hypusine, in the eukaryotic translation initiation factor 5A (eIF5A). J. Biochem. 139, 161–169, 10.1093/jb/mvj034 (2006). 16452303 PMC2494880

[b8] Glick B. R. & Ganoza M. C. Identification of a soluble protein that stimulates peptide bond synthesis. Proc. Natl. Acad. Sci. USA 72, 4257–4260 (1975). 1105576 10.1073/pnas.72.11.4257 PMC388699

[b9] Lassak J., Wilson D. N. & Jung K. Stall no more at polyproline stretches with the translation elongation factors EF-P and IF-5A. Mol. Microbiol. 10.1111/mmi.13233 (2015). 26416626

[b10] Roy H. *et al.* The tRNA synthetase paralog PoxA modifies elongation factor-P with (R)-beta-lysine. Nature Chem. Biol. 7, 667–669, 10.1038/nchembio.632 (2011). 21841797 PMC3177975

[b11] Park J. H. *et al.* Post-translational modification by beta-lysylation is required for activity of Escherichia coli elongation factor P (EF-P). J. Biol. Chem. 287, 2579–2590, 10.1074/jbc.M111.309633 (2012). 22128152 PMC3268417

[b12] Blaha G., Stanley R. E. & Steitz T. A. Formation of the first peptide bond: the structure of EF-P bound to the 70S ribosome. Science 325, 966–970, 10.1126/science.1175800 (2009). 19696344 PMC3296453

[b13] Doerfel L. K. *et al.* EF-P is essential for rapid synthesis of proteins containing consecutive proline residues. Science 339, 85–88, 10.1126/science.1229017 (2013). 23239624

[b14] Ude S. *et al.* Translation elongation factor EF-P alleviates ribosome stalling at polyproline stretches. Science 339, 82–85, 10.1126/science.1228985 (2013). 23239623

[b15] Hersch S. J. *et al.* Divergent protein motifs direct elongation factor P-mediated translational regulation in Salmonella enterica and Escherichia coli. mBio 4, e00180–00113, 10.1128/mBio.00180-13 (2013). 23611909 PMC3638311

[b16] Peil L. *et al.* Distinct XPPX sequence motifs induce ribosome stalling, which is rescued by the translation elongation factor EF-P. Proc. Natl. Acad. Sci. USA 110, 15265–15270, 10.1073/pnas.1310642110 (2013). 24003132 PMC3780873

[b17] Woolstenhulme C. J., Guydosh N. R., Green R. & Buskirk A. R. High-precision analysis of translational pausing by ribosome profiling in bacteria lacking EFP. Cell reports 11, 13–21, 10.1016/j.celrep.2015.03.014 (2015). 25843707 PMC4835038

[b18] Kang H. A. & Hershey J. W. Effect of initiation factor eIF-5A depletion on protein synthesis and proliferation of Saccharomyces cerevisiae. J. Biol. Chem. 269, 3934–3940 (1994). 8307948

[b19] Gregio A. P., Cano V. P., Avaca J. S., Valentini S. R. & Zanelli C. F. eIF5A has a function in the elongation step of translation in yeast. Biochem. Biophys. Res. Com. 380, 785–790, 10.1016/j.bbrc.2009.01.148 (2009). 19338753

[b20] Saini P., Eyler D. E., Green R. & Dever T. E. Hypusine-containing protein eIF5A promotes translation elongation. Nature 459, 118–121, 10.1038/nature08034 (2009). 19424157 PMC3140696

[b21] Gutierrez E. *et al.* eIF5A promotes translation of polyproline motifs. Mol. Cell 51, 35–45, 10.1016/j.molcel.2013.04.021 (2013). 23727016 PMC3744875

[b22] Zuk D. & Jacobson A. A single amino acid substitution in yeast eIF-5A results in mRNA stabilization. Embo. J. 17, 2914–2925 (1998). 9582285 10.1093/emboj/17.10.2914 PMC1170632

[b23] Chatterjee I., Gross S. R., Kinzy T. G. & Chen K. Y. Rapid depletion of mutant eukaryotic initiation factor 5A at restrictive temperature reveals connections to actin cytoskeleton and cell cycle progression. Mol. Gen. Genom.: MGG 275, 264–276, 10.1007/s00438-005-0086-4 (2006). 16408210

[b24] Zanelli C. F. & Valentini S. R. Pkc1 acts through Zds1 and Gic1 to suppress growth and cell polarity defects of a yeast eIF5A mutant. Genetics 171, 1571–1581, 10.1534/genetics.105.048082 (2005). 16157662 PMC1456085

[b25] Rossi D. *et al.* eIF5A has a function in the cotranslational translocation of proteins into the ER. Amino Acids 46, 645–653, 10.1007/s00726-013-1618-6 (2014). 24306454

[b26] Hanauske-Abel H. M. *et al.* Inhibition of the G1-S transition of the cell cycle by inhibitors of deoxyhypusine hydroxylation. Biochim. Biophys. Acta 1221, 115–124 (1994). 8148388 10.1016/0167-4889(94)90003-5

[b27] Ruhl M. *et al.* Eukaryotic initiation factor 5A is a cellular target of the human immunodeficiency virus type 1 Rev activation domain mediating trans- activation. J. Cell. Biol. 123, 1309–1320 (1993). 8253832 10.1083/jcb.123.6.1309 PMC2290910

[b28] Hoque M. *et al.* Inhibition of HIV-1 gene expression by Ciclopirox and Deferiprone, drugs that prevent hypusination of eukaryotic initiation factor 5A. Retrovirol. 6, 90–106, 10.1186/1742-4690-6-90 (2009). PMC2770518 19825182

[b29] Caraglia M. *et al.* The eukaryotic initiation factor 5A is involved in the regulation of proliferation and apoptosis induced by interferon-alpha and EGF in human cancer cells. J. Biochem. 133, 757–765 (2003). 12869532 10.1093/jb/mvg097

[b30] Li C. H., Ohn T., Ivanov P., Tisdale S. & Anderson P. eIF5A promotes translation elongation, polysome disassembly and stress granule assembly. PloS One 5, e9942, 10.1371/journal.pone.0009942 (2010). 20376341 PMC2848580

[b31] Robbins R. D. *et al.* Inhibition of deoxyhypusine synthase enhances islet {beta} cell function and survival in the setting of endoplasmic reticulum stress and type 2 diabetes. J. Biol. Chem. 285, 39943–39952, 10.1074/jbc.M110.170142 (2010). 20956533 PMC3000976

[b32] Memin E. *et al.* Blocking eIF5A modification in cervical cancer cells alters the expression of cancer-related genes and suppresses cell proliferation. Cancer Res. 74, 552–562, 10.1158/0008-5472.CAN-13-0474 (2014). 24220243 PMC4745653

[b33] Fujimura K. *et al.* Eukaryotic Translation Initiation Factor 5A (EIF5A) Regulates Pancreatic Cancer Metastasis by Modulating RhoA and Rho-associated Kinase (ROCK) Protein Expression Levels. J. Biol. Chem. 290, 29907–29919, 10.1074/jbc.M115.687418 (2015). 26483550 PMC4706006

[b34] Nishiki Y. *et al.* Characterization of a novel polyclonal anti-hypusine antibody. SpringerPlus 2, 421, 10.1186/2193-1801-2-421 (2013). 24024105 PMC3765601

[b35] Gosslau A., Jao D. L., Butler R., Liu A. Y. & Chen K. Y. Thermal killing of human colon cancer cells is associated with the loss of eukaryotic initiation factor 5A. J. Cell. Physiol. 219, 485–493, 10.1002/jcp.21696 (2009). 19160416

[b36] Starosta A. L. *et al.* Translational stalling at polyproline stretches is modulated by the sequence context upstream of the stall site. Nucleic Acids Res. 42, 10711–10719, 10.1093/nar/gku768 (2014). 25143529 PMC4176338

[b37] Hersch S. J., Elgamal S., Katz A., Ibba M. & Navarre W. W. Translation initiation rate determines the impact of ribosome stalling on bacterial protein synthesis. J. Biol. Chem. 289, 28160–28171, 10.1074/jbc.M114.593277 (2014). 25148683 PMC4192472

[b38] Hanauske-Abel H. M. *et al.* Detection of a sub-set of polysomal mRNAs associated with modulation of hypusine formation at the G1-S boundary. Proposal of a role for eIF-5A in onset of DNA replication. FEBS Lett. 366, 92–98 (1995). 7789538 10.1016/0014-5793(95)00493-s

[b39] Boisvert F. M. *et al.* A quantitative spatial proteomics analysis of proteome turnover in human cells. Mol. Cell. Proteomics 11, M111 011429, 10.1074/mcp.M111.011429 (2012). 21937730 PMC3316722

[b40] Hetz C. The unfolded protein response: controlling cell fate decisions under ER stress and beyond. Nature Rev. Mol. Cell Biol 13, 89–102, 10.1038/nrm3270 (2012). 22251901

[b41] Frigieri M. C., Joao Luiz M. V., Apponi L. H., Zanelli C. F. & Valentini S. R. Synthetic lethality between eIF5A and Ypt1 reveals a connection between translation and the secretory pathway in yeast. Mol. Gen. Genomics: MGG 280, 211–221, 10.1007/s00438-008-0357-y (2008). 18568365

[b42] Duan X. *et al.* A straightforward and highly efficient precipitation/on-pellet digestion procedure coupled with a long gradient nano-LC separation and Orbitrap mass spectrometry for label-free expression profiling of the swine heart mitochondrial proteome. J. Proteome Res. 8, 2838–2850, 10.1021/pr900001t (2009). 19290621 PMC2734143

[b43] Tang W. H., Shilov I. V. & Seymour S. L. Nonlinear fitting method for determining local false discovery rates from decoy database searches. J. Proteome Res. 7, 3661–3667, 10.1021/pr070492f (2008). 18700793

[b44] Pascovici D. *et al.* Combining protein ratio p-values as a pragmatic approach to the analysis of multirun iTRAQ experiments. J. Proteome Res. 14, 738–746, 10.1021/pr501091e (2015). 25495031

[b45] R Core Team. R: A language and environment for statistical computing. (2015) Available at: https://www.R-project.org/. (Accessed: 4th November 2015).

[b46] Mi H., Muruganujan A. & Thomas P. D. PANTHER in 2013: modeling the evolution of gene function, and other gene attributes, in the context of phylogenetic trees. Nucleic Acids Res. 41, D377–386, 10.1093/nar/gks1118 (2013). 23193289 PMC3531194

[b47] Shannon P. *et al.* Cytoscape: a software environment for integrated models of biomolecular interaction networks. Genome Res. 13, 2498–2504, 10.1101/gr.1239303 (2003). 14597658 PMC403769

[b48] Bindea G. *et al.* ClueGO: a Cytoscape plug-in to decipher functionally grouped gene ontology and pathway annotation networks. Bioinformatics 25, 1091–1093, 10.1093/bioinformatics/btp101 (2009). 19237447 PMC2666812

[b49] Bindea G., Galon J. & Mlecnik B. CluePedia Cytoscape plugin: pathway insights using integrated experimental and in silico data. Bioinformatics 29, 661–663, 10.1093/bioinformatics/btt019 (2013). 23325622 PMC3582273

[b50] Mandal A., Mandal S. & Park M. H. Genome-wide analyses and functional classification of proline repeat-rich proteins: potential role of eIF5A in eukaryotic evolution. PloS One 9, e111800, 10.1371/journal.pone.0111800 (2014). 25364902 PMC4218817

