# Supplementary material for: Global quantitative proteomics reveal up-regulation of endoplasmic reticulum stress response proteins upon depletion of eIF5A in HeLa cells
Source: Sci Rep. 2016 May 16;6:25795. doi: 10.1038/srep25795 (PMC4867578; doi:10.1038/srep25795)
Supplement: Supplementary Information [file srep25795-s1.pdf]

Title: Global quantitative proteomic analyses reveal up-regulation of endoplasmic reticulum stress response proteins upon depletion of eIF5A in HeLa cells

Authors: Ajeet Mandal[‡], Swati Mandal[‡] and Myung Hee Park[*]

Supplementary Table S1. Alphabetical listing of 972 unique proteins commonly identified from three independent iTRAQ experiments. The ratios of each protein in Ad-eIF5A-shRNA-transduced cells *vs* Ad-scramble-shRNA-transduced cells are shown at 72, 96 and 120h of transduction. The geometric mean of ratios from the three iTRAQ data sets is given at each time point and those ratios with p-value ≤0.05 are indicated by an asterisk (*) superscript. The computed number of polyproline motifs (PPP) is given for each protein.

#	Accession	Gene symbol	Gene/Protein description	Protein ratio (72h)	Protein ratio (96h)	Protein ratio (120h)	# of PPP-units
1	gi|109148542	AARS	alanine--tRNA ligase, cytoplasmic	1.15	0.93*	0.2*	0
2	gi|38569417	AARS2	alanine--tRNA ligase, mitochondrial	0.93	1.11	0.29*	0
3	gi|69354671	ABCF1	ATP-binding cassette sub-family F member 1 isoform a	1.59	0.80	0.52	1
4	gi|8923001	ABHD10	mycophenolic acid acyl-glucuronide esterase, mitochondrial isoform 1 precursor	2.12	1.51	0.86	0
5	gi|167614485	ACAA2	3-ketoacyl-CoA thiolase, mitochondrial	1.21	1.24*	0.29*	0
6	gi|4557231	ACADM	medium-chain specific acyl-CoA dehydrogenase, mitochondrial isoform a precursor	0.97	0.71	0.19*	0
7	gi|4557237	ACAT1	acetyl-CoA acetyltransferase, mitochondrial precursor	1.11	1.06*	0.65	0
8	gi|148539872	ACAT2	acetyl-CoA acetyltransferase, cytosolic isoform 1	0.92	0.78	0.59	0
9	gi|38569421	ACLY	ATP-citrate synthase isoform 1	0.97*	0.71	0.5*	2
10	gi|8659555	ACO1	cytoplasmic aconitate hydratase	1.17	1.80	0.92	0
11	gi|4501867	ACO2	aconitate hydratase, mitochondrial precursor	1.14	1.48	0.67	0
12	gi|8923812	ACOT13	acyl-coenzyme A thioesterase 13 isoform 1	0.72	1.05*	0.73*	0

13	gi|42794752	ACSL3	long-chain-fatty-acid--CoA ligase 3	1.08	0.60	1.15	0
14	gi|4501887	ACTG1	actin, cytoplasmic 2	1.37	0.73	0.5*	0
15	gi|4757718	ACTL6A	actin-like protein 6A isoform 1	1.20	0.83	1.39	0
16	gi|4501891	ACTN1	alpha-actinin-1 isoform b	1.09*	1.32*	0.66	0
17	gi|12025678	ACTN4	alpha-actinin-4	0.99	1.32	0.37*	0
18	gi|5031569	ACTR1A	alpha-centractin	0.55	0.80	0.99	0
19	gi|11342680	ACTR1B	beta-centractin	0.71	0.73	1.18	0
20	gi|5031573	ACTR3	actin-related protein 3 isoform 1	0.85	1.19	0.66	0
21	gi|4501901	ACY1	aminoacylase-1 isoform a	1.41	1.68	0.79	0
22	gi|71565154	ADH5	alcohol dehydrogenase class-3	0.78	0.99	0.59	0
23	gi|32484975	ADK	adenosine kinase isoform b	0.59	0.55	0.36*	0
24	gi|34577063	ADSS	adenylosuccinate synthetase isozyme 2	1.04	1.18	0.84	0
25	gi|116734847	AGL	glycogen debranching enzyme isoform 1	0.90	0.76	0.54*	0
26	gi|4501993	AGPS	alkyldihydroxyacetonephosphate synthase, peroxisomal precursor	1.23	0.58	0.42*	0
27	gi|262359929	AHCTF1	protein ELYS	0.77	0.91	1.01	2
28	gi|9951915	AHCY	adenosylhomocysteinase isoform 1	0.77	1.17	0.54*	0
29	gi|61743954	AHNAK	neuroblast differentiation-associated protein AHNAK isoform 1	0.93	0.98*	0.32*	0
30	gi|156766050	AHNAK2	protein AHNAK2	0.84	0.83	0.84	0
31	gi|4757732	AIFM1	apoptosis-inducing factor 1, mitochondrial isoform AIF precursor	0.82	1.22	0.57	0
32	gi|11125770	AIMP2	aminoacyl tRNA synthase complex-interacting multifunctional protein 2	1.00	0.69	0.88	0

33	gi|4502011	AK1	adenylate kinase isoenzyme 1	1.27	0.83	0.33*	0
34	gi|8051579	AK4	adenylate kinase 4, mitochondrial	1.23	1.06	0.75	0
35	gi|4502049	AKR1B1	aldose reductase	1.17	0.86	0.43	0
36	gi|62912457	ALDH18A1	delta-1-pyrroline-5-carboxylate synthase isoform 2	1.03	0.75	0.77	0
37	gi|115387104	ALDH9A1	4-trimethylaminobutyraldehyde dehydrogenase	0.70	1.06	0.99	0
38	gi|4885063	ALDOC	fructose-bisphosphate aldolase C	1.62	1.44	0.47	0
39	gi|238776833	ALYREF	THO complex subunit 4	1.11	1.47	0.60	0
40	gi|5453880	ANP32A	acidic leucine-rich nuclear phosphoprotein 32 family member A	0.48	0.80	0.55	0
41	gi|5454088	ANP32B	acidic leucine-rich nuclear phosphoprotein 32 family member B	0.71*	0.71*	0.4*	0
42	gi|4502101	ANXA1	annexin A1	0.53	1.14	1*	0
43	gi|4557317	ANXA11	annexin A11 isoform 1	0.84	0.74	2.13*	4
44	gi|4826643	ANXA3	annexin A3	1.23	0.39	1.21	0
45	gi|4502105	ANXA4	annexin A4	0.63	0.70	1.48	0
46	gi|4502107	ANXA5	annexin A5	1.27	0.83	1.65*	0
47	gi|71773329	ANXA6	annexin A6 isoform 1	0.76*	0.66	1.8*	0
48	gi|4809279	ANXA7	annexin A7 isoform 2	0.92*	0.84	1.69*	0
49	gi|71773106	AP2B1	AP-2 complex subunit beta isoform a	0.50	0.81	1.07	0
50	gi|68799814	AP2M1	AP-2 complex subunit mu isoform b	1.39	0.88	0.84	0
51	gi|23510451	APEH	acylamino-acid-releasing enzyme	0.75	0.70	0.29	1
52	gi|18375501	APEX1	DNA-(apurinic or apyrimidinic site) lyase	0.37	0.76	0.67	0

53	gi|24308201	APMAP	adipocyte plasma membrane-associated protein	1.14	1.33	2.14	0
54	gi|91984773	APOA1BP	NAD(P)H-hydrate epimerase precursor	1.40	0.88	0.67	0
55	gi|4502171	APRT	adenine phosphoribosyltransferase isoform a	0.78	0.63	0.3*	0
56	gi|11863154	ARCN1	coatomer subunit delta isoform 1	1.18	1.18	1.27	0
57	gi|4757768	ARHGDIA	rho GDP-dissociation inhibitor 1 isoform a	1.01	1.21	0.77	0
58	gi|253735778	ARHGEF2	rho guanine nucleotide exchange factor 2 isoform 2	0.73	1.24	1.38	0
59	gi|5031595	ARPC4	actin-related protein 2/3 complex subunit 4 isoform a	1.03	0.51	0.89	0
60	gi|50428938	ASNA1	ATPase ASNA1	0.66	0.91	0.74	0
61	gi|16950633	ASS1	argininosuccinate synthase	1.29	0.57	0.3*	0
62	gi|283436222	ATAD3A	ATPase family AAA domain-containing protein 3A isoform 2	0.69	0.77	1.08	1
63	gi|19526773	ATG3	ubiquitin-like-conjugating enzyme ATG3 isoform 1	0.94	1.18	0.63	1
64	gi|20127454	ATIC	bifunctional purine biosynthesis protein PURH	0.82	1.10	0.38*	0
65	gi|45827806	ATL3	atlastin-3 isoform 1	1.29	1.17	1.19	0
66	gi|48255959	ATP2B4	plasma membrane calcium-transporting ATPase 4 isoform 4a	1.06	0.90	1.22	0
67	gi|4757810	ATP5A1	ATP synthase subunit alpha, mitochondrial isoform a precursor	0.88*	1.84*	2.12*	0
68	gi|32189394	ATP5B	ATP synthase subunit beta, mitochondrial precursor	0.81*	1.22	2.15*	0
69	gi|50345988	ATP5C1	ATP synthase subunit gamma, mitochondrial isoform L (liver) precursor	0.74	0.69	1.02	0
70	gi|5453559	ATP5H	ATP synthase subunit d, mitochondrial isoform a	0.62	1.73	3.53*	0
71	gi|51479156	ATP5L	ATP synthase subunit g, mitochondrial	0.53	0.88	2.16	0
72	gi|4502303	ATP5O	ATP synthase subunit O, mitochondrial precursor	0.88	1.57*	2.24*	0

73	gi|19913424	ATP6V1A	V-type proton ATPase catalytic subunit A	0.80	1.15	0.89	0
74	gi|19913428	ATP6V1B2	V-type proton ATPase subunit B, brain isoform	2.23	0.51	1.23	0
75	gi|7106299	ATXN10	ataxin-10 isoform 1	0.56	1.46	1.01	1
76	gi|14043024	BAG3	BAG family molecular chaperone regulator 3	0.83	1.99*	0.69	2
77	gi|30795231	BASP1	brain acid soluble protein 1	3.45*	2.53*	0.89*	0
78	gi|61742797	BCAM	basal cell adhesion molecule isoform 2 precursor	1.03	1.04	1.01	0
79	gi|4557367	BLMH	bleomycin hydrolase	1.72	0.97	0.46	0
80	gi|4502419	BLVRB	flavin reductase (NADPH)	0.88	0.43	0.47	0
81	gi|7705638	BOLA1	bolA-like protein 1 precursor	1.32	1.81	0.67	0
82	gi|21327667	BOP1	ribosome biogenesis protein BOP1	0.93	0.77	0.76	1
83	gi|116812595	BPNT1	3'(2'),5'-bisphosphate nucleotidase 1 isoform 1	0.99	1.10	0.85	0
84	gi|38372925	BSG	basigin isoform 2	1.41	1.32	1.60	0
85	gi|83641885	BTF3	transcription factor BTF3 isoform A	0.68	1.05	0.48	1
86	gi|333470721	BZW1	basic leucine zipper and W2 domain-containing protein 1 isoform 3	0.99	0.53	0.45*	0
87	gi|7661744	BZW2	basic leucine zipper and W2 domain-containing protein 2	1.20	0.77	0.37	0
88	gi|145275185	C12orf10	UPF0160 protein MYG1, mitochondrial precursor	1.07*	1.05	0.3*	2
89	gi|19923951	C12orf57	protein C10 isoform 1	1.22	1.37	0.52	0
90	gi|33457348	C19orf10	UPF0556 protein C19orf10 precursor	0.77	1.41	2.18	0
91	gi|4502491	C1QBP	complement component 1 Q subcomponent-binding protein, mitochondrial precursor	0.77	1.10	2.13*	0
92	gi|296531406	C21orf33	ES1 protein homolog, mitochondrial isoform Ia precursor	0.82	1.02	0.93	0

93	gi|7656952	CACYBP	calcyclin-binding protein isoform 1	0.64	0.90	0.46	0
94	gi|18105007	CAD	CAD protein isoform 1	0.79	0.57	0.42*	3
95	gi|4502549	CALM1	calmodulin isoform 2	1.65	0.95	0.82	0
96	gi|4757900	CALR	calreticulin precursor	0.65	1.64*	4.09*	0
97	gi|21361794	CAND1	cullin-associated NEDD8-dissociated protein 1	0.44	0.71	1.12	0
98	gi|10716563	CANX	calnexin precursor	1.49	1.35	2.63*	0
99	gi|5453595	CAP1	adenylyl cyclase-associated protein 1	1.24*	1.02	0.65*	3
100	gi|63252913	CAPG	macrophage-capping protein isoform 1	1.44	0.77	0.31*	0
101	gi|157389005	CAPN2	calpain-2 catalytic subunit isoform 1	0.72	0.76	0.73	1
102	gi|4502565	CAPNS1	calpain small subunit 1 isoform 1	0.23	1.71	2.15	1
103	gi|42558250	CAPRIN1	caprin-1 isoform 1	1.25	0.90	0.66	1
104	gi|5453597	CAPZA1	F-actin-capping protein subunit alpha-1	0.95	1.08	0.90	0
105	gi|62240992	CARS	cysteine--tRNA ligase, cytoplasmic isoform c	0.88	0.64	0.98	0
106	gi|109637759	CAST	calpastatin isoform f	1.27	0.85	0.66	0
107	gi|4557014	CAT	catalase	1.54	1.16	1.86*	0
108	gi|4557415	CBS	cystathionine beta-synthase	0.64	0.80	0.25*	0
109	gi|15082258	CBX3	chromobox protein homolog 3	1.15	0.90	0.52	0
110	gi|6912292	CBX5	chromobox protein homolog 5	0.50	1.29	0.34	0
111	gi|24432106	CCAR2	cell cycle and apoptosis regulator protein 2	1.29	0.60	0.50	3
112	gi|171906582	CCDC47	coiled-coil domain-containing protein 47 precursor	0.62	1.14	2.75*	0

113	gi|46852390	CCDC6	coiled-coil domain-containing protein 6	0.71	0.94	0.39	4
114	gi|5453603	CCT2	T-complex protein 1 subunit beta isoform 1	0.90	0.68*	1.11	0
115	gi|63162572	CCT3	T-complex protein 1 subunit gamma isoform a	1.06	0.57	1.40	0
116	gi|38455427	CCT4	T-complex protein 1 subunit delta isoform a	0.99*	0.92	1.54*	0
117	gi|24307939	CCT5	T-complex protein 1 subunit epsilon isoform a	1.29*	0.8*	1.25	0
118	gi|4502643	CCT6A	T-complex protein 1 subunit zeta isoform a	1.05	0.61	1.32	0
119	gi|5453607	CCT7	T-complex protein 1 subunit eta isoform a	1.05	0.62*	1.72*	0
120	gi|48762932	CCT8	T-complex protein 1 subunit theta isoform 1	0.87	1.22	1.92*	0
121	gi|227430301	CD109	CD109 antigen isoform 2 preproprotein	1.03	0.89	2.05	0
122	gi|11321634	CD2AP	CD2-associated protein	0.85	1.41	1.09	2
123	gi|168693643	CD55	complement decay-accelerating factor isoform 2 precursor	1.78	1.11	0.9*	1
124	gi|11386157	CDA	cytidine deaminase	0.92	1.49	0.70	0
125	gi|5901922	CDC37	hsp90 co-chaperone Cdc37	1.39	0.83	0.34*	0
126	gi|4757952	CDC42	cell division control protein 42 homolog isoform 1 precursor	2.16	0.76	0.47	0
127	gi|13786127	CDC42EP4	cdc42 effector protein 4	0.95	0.75	1.02	0
128	gi|11067747	CDC5L	cell division cycle 5-like protein	1.12	0.84	0.58	0
129	gi|4502709	CDK1	cyclin-dependent kinase 1 isoform 1	1.09	1.01	0.55	0
130	gi|4502749	CDKN2A	cyclin-dependent kinase inhibitor 2A isoform p16INK4a	0.95	0.29*	0.36	0
131	gi|8923710	CDV3	protein CDV3 homolog isoform b	0.93	0.83	0.12*	0
132	gi|42542392	CEBPZ	CCAAT/enhancer-binding protein zeta	0.96	1.08	3.18	0

133	gi|5031635	CFL1	cofilin-1	1.38	2.1*	0.16*	0
134	gi|7705851	CHCHD2	coiled-coil-helix-coiled-coil-helix domain-containing protein 2 precursor	0.90	0.63	0.19	0
135	gi|221316566	CHORDC1	cysteine and histidine-rich domain-containing protein 1 isoform a	0.81	0.67	0.27	0
136	gi|19920317	CKAP4	cytoskeleton-associated protein 4	1.67	2.82*	4.76*	2
137	gi|21536286	CKB	creatine kinase B-type	1.44	0.82	0.19*	0
138	gi|10334859	CKMT1A	creatine kinase U-type, mitochondrial precursor	0.94	0.98	0.35*	0
139	gi|14251209	CLIC1	chloride intracellular channel protein 1	0.60	0.73	0.90	0
140	gi|4502891	CLNS1A	methylosome subunit pICln isoform a	0.88	1.00	0.66	1
141	gi|5174419	CLPP	ATP-dependent Clp protease proteolytic subunit, mitochondrial precursor	1.06	2.08	1.02	0
142	gi|6005993	CLTA	clathrin light chain A isoform b	1.24	1.32	2.12*	0
143	gi|4758012	CLTC	clathrin heavy chain 1 isoform 1	0.65*	0.88	2.92*	0
144	gi|355594753	CLU	clusterin preproprotein	0.95	1.11	1.36	0
145	gi|20270371	CMBL	carboxymethylenebutenolidase homolog	1.10	1.23	0.49	0
146	gi|7706497	CMPK1	UMP-CMP kinase isoform a	1.23	0.94	0.32	0
147	gi|271398239	CNDP2	cytosolic non-specific dipeptidase isoform 1	1.12	1.06	0.75*	0
148	gi|7657176	CNPY2	protein canopy homolog 2 isoform 1 precursor	0.82	1.80	1.99	0
149	gi|31377697	COLGALT1	procollagen galactosyltransferase 1 precursor	0.94	3.25*	4.31*	0
150	gi|7705369	COPB1	coatomer subunit beta	1.09	1.02	1.46	0
151	gi|4758032	COPB2	coatomer subunit beta'	0.62	0.61	0.75	0
152	gi|31542319	COPE	coatomer subunit epsilon isoform a	0.99*	1.44	1.55	0

153	gi|11559929	COPG1	coatomer subunit gamma-1	0.94	0.72	1.01	0
154	gi|109134349	COPG2	coatomer subunit gamma-2 isoform 1	0.53	0.47	0.87	0
155	gi|38373690	COPS4	COP9 signalosome complex subunit 4 isoform 1	1.10	0.88	1.06	0
156	gi|5729779	COPS8	COP9 signalosome complex subunit 8 isoform 1	1.17	0.68	0.69	0
157	gi|7706337	COPZ1	coatomer subunit zeta-1 isoform 1	0.62	0.72	2.62	0
158	gi|21624607	COTL1	coactosin-like protein	0.91	0.83	0.40	0
159	gi|4502981	COX4I1	cytochrome c oxidase subunit 4 isoform 1, mitochondrial precursor	1.24	1.45	1.54	0
160	gi|190885499	COX5A	cytochrome c oxidase subunit 5A, mitochondrial precursor	0.42	1.35*	2.63*	0
161	gi|4502985	COX6B1	cytochrome c oxidase subunit 6B1	0.92*	0.87	0.74	0
162	gi|4503015	CPNE3	copine-3	1.03	1.08	0.86	0
163	gi|41393599	CPOX	oxygen-dependent coproporphyrinogen-III oxidase, mitochondrial precursor	0.68	0.79	0.70	0
164	gi|34101288	CPSF2	cleavage and polyadenylation specificity factor subunit 2	1.10	0.78	1.10	0
165	gi|162329583	CPSF6	cleavage and polyadenylation specificity factor subunit 6 isoform 1	1.04	1.21	0.61	14
166	gi|41327712	CRK	adapter molecule crk isoform a	1.39	0.77	0.37	1
167	gi|4885153	CRKL	crk-like protein	1.57	1.85	0.25*	0
168	gi|38327625	CS	citrate synthase, mitochondrial precursor	1.86*	1.18	2.1*	0
169	gi|4758086	CSRP1	cysteine and glycine-rich protein 1 isoform 1	1.15	0.86	0.47	0
170	gi|4503117	CSTB	cystatin-B	0.92	1.04	0.14*	0
171	gi|4557493	CSTF2	cleavage stimulation factor subunit 2 isoform 2	0.74	1.15	0.49	1
172	gi|55770844	CTNNA1	catenin alpha-1 isoform 1	0.98	0.86	0.64	0

173	gi|4503139	CTSB	cathepsin B preproprotein	0.88	2.11	1.97*	0
174	gi|189083844	CTSC	dipeptidyl peptidase 1 isoform a preproprotein	0.82	1.04	0.47	0
175	gi|4503143	CTSD	cathepsin D preproprotein	1.03	0.70	2.28*	1
176	gi|22538442	CTSZ	cathepsin Z preproprotein	1.04	0.99	0.63	0
177	gi|20357552	CTTN	src substrate cortactin isoform a	0.90	0.91	0.32*	0
178	gi|121114302	CUL4B	cullin-4B isoform 2	0.66	0.70	1.59	0
179	gi|21359867	CYC1	cytochrome c1, heme protein, mitochondrial precursor	0.91	0.87	1.02	0
180	gi|11128019	CYCS	cytochrome c	0.77	1.02	0.67	0
181	gi|294997282	DAG1	dystroglycan preproprotein	1.59	1.21	1.48	4
182	gi|20149621	DAK	bifunctional ATP-dependent dihydroxyacetone kinase/FAD-AMP lyase (cyclizing)	2.16	3.52	0.33*	1
183	gi|45439306	DARS	aspartate--tRNA ligase, cytoplasmic isoform 1	1.34	0.81	0.81	0
184	gi|25470890	DAZAP1	DAZ-associated protein 1 isoform a	1.00	0.99	0.46	9
185	gi|56549113	DBR1	lariat debranching enzyme	0.91	0.96	1.47	0
186	gi|5453629	DCTN2	dynactin subunit 2 isoform 1	0.7*	0.80	0.76	0
187	gi|6912328	DDAH1	N(G),N(G)-dimethylarginine dimethylaminohydrolase 1 isoform 1	1.87	0.92	0.59	0
188	gi|148529014	DDB1	DNA damage-binding protein 1	0.53*	0.89	0.84	0
189	gi|62955833	DDI2	protein DDI1 homolog 2	0.36*	1.39	0.63	0
190	gi|4503291	DDT	D-dopachrome decarboxylase	0.62	0.97	0.55	0
191	gi|4826686	DDX1	ATP-dependent RNA helicase DDX1	0.77	0.56	0.31*	0
192	gi|50659095	DDX21	nucleolar RNA helicase 2 isoform 1	0.78*	0.48*	0.54*	0

193	gi|21040371	DDX39A	ATP-dependent RNA helicase DDX39A	0.73	0.76	0.65	0
194	gi|4758112	DDX39B	spliceosome RNA helicase DDX39B	1.49	0.72	0.31*	0
195	gi|41327773	DDX46	probable ATP-dependent RNA helicase DDX46 isoform 2	0.92	0.98	0.65	0
196	gi|4758138	DDX5	probable ATP-dependent RNA helicase DDX5	0.79	1.18	1.21	0
197	gi|4503301	DECR1	2,4-dienoyl-CoA reductase, mitochondrial precursor	1.25	1.08	1.64*	0
198	gi|4503249	DEK	protein DEK isoform 1	0.78	0.81	0.52	0
199	gi|27501446	DENR	density-regulated protein	0.72	0.88*	0.30	0
200	gi|4758148	DFFA	DNA fragmentation factor subunit alpha isoform 1	0.80	0.97	0.58	0
201	gi|68509926	DHX15	pre-mRNA-splicing factor ATP-dependent RNA helicase DHX15	1.02	1.45	0.71	1
202	gi|100913206	DHX9	ATP-dependent RNA helicase A	0.81*	0.80	2.29*	4
203	gi|9845297	DIABLO	diablo homolog, mitochondrial isoform 1 precursor	1.08	1.03	0.80	0
204	gi|55749758	DIP2B	disco-interacting protein 2 homolog B	1.00	2.02	1.21	0
205	gi|31711992	DLAT	dihydrolipoyllysine-residue acetyltransferase component of pyruvate dehydrogenase complex, mitochondrial precursor	1.26	2.00	1.30	2
206	gi|91199540	DLD	dihydrolipoyl dehydrogenase, mitochondrial isoform 1 precursor	1.02	1.44*	2.42*	0
207	gi|19923748	DLST	dihydrolipoyllysine-residue succinyltransferase component of 2-oxoglutarate dehydrogenase complex, mitochondrial isoform 1 precursor	0.81	0.94	1.53	1
208	gi|4504511	DNAJA1	dnaJ homolog subfamily A member 1	1.01	0.79	1.49	0
209	gi|5031741	DNAJA2	dnaJ homolog subfamily A member 2	1.06	0.98	1.19	0
210	gi|5453690	DNAJB1	dnaJ homolog subfamily B member 1 isoform 1	1.98	0.94	2.15	0
211	gi|7706495	DNAJB11	dnaJ homolog subfamily B member 11 precursor	0.85	1.19	1.80	0
212	gi|6631085	DNAJB4	dnaJ homolog subfamily B member 4	1.42	0.69	0.38	0

213	gi|262206280	DNAJC7	dnaJ homolog subfamily C member 7 isoform 1	0.87	1.04	0.46	0
214	gi|112293277	DNAJC8	dnaJ homolog subfamily C member 8	0.56	0.79	0.72	0
215	gi|27597059	DNAJC9	dnaJ homolog subfamily C member 9	0.85	0.79	0.77	0
216	gi|4503349	DNASE2	deoxyribonuclease-2-alpha precursor	0.97	1.07	1.31	1
217	gi|171460916	DNM1L	dynamin-1-like protein isoform 2	0.78	0.90	0.69	0
218	gi|5454002	DNPH1	2'-deoxynucleoside 5'-phosphate N-hydrolase 1 isoform 1	0.88	0.96	0.52	0
219	gi|54633315	DNTTIP2	deoxynucleotidyltransferase terminal-interacting protein 2	1.19	0.64	0.44	0
220	gi|117190262	DPH5	diphthine methyl ester synthase isoform a	1.74	1.05	1.27	0
221	gi|116534898	DSG2	desmoglein-2 preproprotein	1.23	1.25	1.06	0
222	gi|58530840	DSP	desmoplakin isoform I	0.97*	1.07	0.33*	1
223	gi|33350932	DYNC1H1	cytoplasmic dynein 1 heavy chain 1	0.68	0.76	1.37*	1
224	gi|422398894	DYNC1I2	cytoplasmic dynein 1 intermediate chain 2 isoform 4	0.80	0.89	0.96	1
225	gi|166795297	DYNC1LI1	cytoplasmic dynein 1 light intermediate chain 1	0.76	0.55	1.22	0
226	gi|70995211	ECH1	delta(3,5)-Delta(2,4)-dienoyl-CoA isomerase, mitochondrial precursor	1.76	1.68	2.02*	0
227	gi|194097323	ECHS1	enoyl-CoA hydratase, mitochondrial	0.96	0.91	1.97*	0
228	gi|62530384	ECI1	enoyl-CoA delta isomerase 1, mitochondrial isoform 1 precursor	0.88	0.74	1.05	0
229	gi|45827771	EDC4	enhancer of mRNA-decapping protein 4	1.11	0.75	0.31	0
230	gi|4503453	EDF1	endothelial differentiation-related factor 1 isoform alpha	1.54	0.76	0.21*	0
231	gi|55770888	EEA1	early endosome antigen 1	0.66	0.87	1.87*	0
232	gi|4503471	EEF1A1	elongation factor 1-alpha 1	1.13*	0.68*	0.25*	0

233	gi|4503477	EEF1B2	elongation factor 1-beta	1.15	1.52*	0.34	1
234	gi|304555581	EEF1D	elongation factor 1-delta isoform 1	1.51*	1.44	0.54	0
235	gi|4503481	EEF1G	elongation factor 1-gamma	1.00	0.66	0.67	0
236	gi|4503483	EEF2	elongation factor 2	1.57*	0.79*	0.61*	0
237	gi|20149675	EFHD2	EF-hand domain-containing protein D2	1.16	1.21	1.20	0
238	gi|4503499	EIF1AX	eukaryotic translation initiation factor 1A, X-chromosomal	0.88	1.01	0.47	0
239	gi|54873624	EIF2A	eukaryotic translation initiation factor 2A	1.05	1.07	0.82	0
240	gi|4503509	EIF3A	eukaryotic translation initiation factor 3 subunit A	0.87	0.44*	0.77*	1
241	gi|33239445	EIF3B	eukaryotic translation initiation factor 3 subunit B	0.91	0.91	0.94	1
242	gi|4503525	EIF3C	eukaryotic translation initiation factor 3 subunit C isoform a	0.85	1.10	1.39	0
243	gi|4503523	EIF3D	eukaryotic translation initiation factor 3 subunit D	1.29	0.90	0.73	0
244	gi|4503519	EIF3F	eukaryotic translation initiation factor 3 subunit F	0.78	1.05	1.93*	0
245	gi|4503515	EIF3H	eukaryotic translation initiation factor 3 subunit H	0.79	0.87	1.96*	0
246	gi|4503513	EIF3I	eukaryotic translation initiation factor 3 subunit I	0.57	1.00	1.21	0
247	gi|83281438	EIF3J	eukaryotic translation initiation factor 3 subunit J isoform 1	1.13	1.06	0.89	0
248	gi|10801345	EIF3K	eukaryotic translation initiation factor 3 subunit K isoform 1	1.56	0.89	1.15	0
249	gi|7705433	EIF3L	eukaryotic translation initiation factor 3 subunit L isoform 1	1.13	0.63	0.90	0
250	gi|23397429	EIF3M	eukaryotic translation initiation factor 3 subunit M isoform 1	1.52	0.75	1.66	0
251	gi|4503529	EIF4A1	eukaryotic initiation factor 4A-I isoform 1	0.93	0.44	0.37	0
252	gi|7661920	EIF4A3	eukaryotic initiation factor 4A-III	0.59	0.81	0.47	0

253	gi|50053795	EIF4B	eukaryotic translation initiation factor 4B isoform 2	1.13	1.09	0.29	1
254	gi|289577080	EIF4G2	eukaryotic translation initiation factor 4 gamma 2 isoform 1	0.78	0.44	0.62	1
255	gi|21361337	EIF5	eukaryotic translation initiation factor 5	0.58*	0.42	0.16	3
256	gi|4503545	EIF5A	eukaryotic translation initiation factor 5A-1 isoform B	0.2*	0.17*	0.18*	0
257	gi|84043963	EIF5B	eukaryotic translation initiation factor 5B	0.91	0.64	0.79	2
258	gi|4504771	EIF6	eukaryotic translation initiation factor 6 isoform a	0.88	1.05	0.28	0
259	gi|260166622	ELAC2	zinc phosphodiesterase ELAC protein 2 isoform 2	0.83	0.80	0.77	0
260	gi|38201714	ELAVL1	ELAV-like protein 1	1.24	0.48	0.59	0
261	gi|194328699	EMG1	ribosomal RNA small subunit methyltransferase NEP1	0.80	0.77	0.21	0
262	gi|4503571	ENO1	alpha-enolase isoform 1	1.23	0.68	0.33*	0
263	gi|4503579	EPB41L2	band 4.1-like protein 2 isoform a	1.04	0.94	0.46	1
264	gi|62241042	EPRS	bifunctional glutamate/proline--tRNA ligase	1.03	0.56*	0.53	0
265	gi|6005721	ERLIN2	erlin-2 isoform 1	1.01	1.40	2.79	0
266	gi|5803013	ERP29	endoplasmic reticulum resident protein 29 isoform 1 precursor	1.33	1.91*	2.74*	0
267	gi|52487191	ERP44	endoplasmic reticulum resident protein 44 precursor	0.92	1.31	2.47*	0
268	gi|4503607	ETFA	electron transfer flavoprotein subunit alpha, mitochondrial isoform a	0.88	0.86	0.13*	0
269	gi|7705612	EXOSC1	exosome complex component CSL4	0.99	1.01	0.90	0
270	gi|19923403	EXOSC2	exosome complex component RRP4 isoform 1	0.92	0.64	0.69*	0
271	gi|9506689	EXOSC4	exosome complex component RRP41	0.79	1.27	1.19	0
272	gi|189083688	EXOSC7	exosome complex component RRP42	1.15	0.92*	0.94	0

273	gi|21614499	EZR	ezrin	1.63*	0.80	0.32*	2
274	gi|5901948	FAF1	FAS-associated factor 1	1.02	1.22	0.68	1
275	gi|24797106	FAF2	FAS-associated factor 2	1.10	0.63	0.46	1
276	gi|16757970	FAM129A	protein Niban	1.60	0.64	0.42*	1
277	gi|4758220	FAM50A	protein FAM50A	1.08	1.01	0.36	0
278	gi|4758340	FARSA	phenylalanine--tRNA ligase alpha subunit	0.77	1.31	0.86	1
279	gi|124028525	FARSB	phenylalanine--tRNA ligase beta subunit	1.07	1.26	0.88	0
280	gi|41872631	FASN	fatty acid synthase	1.07*	0.63*	0.68	1
281	gi|12056465	FBL	rRNA 2'-O-methyltransferase fibrillarin	0.66	0.43	1.18	1
282	gi|4503685	FDPS	farnesyl pyrophosphate synthase isoform a	1.40	0.63	0.53	0
283	gi|4758356	FEN1	flap endonuclease 1	0.63	0.54	0.57	0
284	gi|19743875	FH	fumarate hydratase, mitochondrial	1.03	0.87	1.86*	0
285	gi|192448443	FKBP10	peptidyl-prolyl cis-trans isomerase FKBP10 precursor	1.34	1.69	2.02*	0
286	gi|150010552	FKBP15	FK506-binding protein 15	0.47	0.80	0.33	1
287	gi|4503725	FKBP1A	peptidyl-prolyl cis-trans isomerase FKBP1A isoform a	1.28	1.59	0.40	0
288	gi|17149842	FKBP2	peptidyl-prolyl cis-trans isomerase FKBP2 precursor	1.52	0.90	1.65	0
289	gi|4503729	FKBP4	peptidyl-prolyl cis-trans isomerase FKBP4	1.09	1.03*	0.49	0
290	gi|4758384	FKBP5	peptidyl-prolyl cis-trans isomerase FKBP5 isoform 1	0.65	0.69	0.90	0
291	gi|52630440	FKBP8	peptidyl-prolyl cis-trans isomerase FKBP8 isoform 1	1.00	0.73	0.42	0
292	gi|33469985	FKBP9	peptidyl-prolyl cis-trans isomerase FKBP9 isoform 1 precursor	1.03	1.41	1.61*	1

293	gi|5031699	FLOT1	flotillin-1	0.81	0.87*	1.82*	0
294	gi|94538362	FLOT2	flotillin-2	0.69	0.82	4.47*	0
295	gi|34147540	FLYWCH2	FLYWCH family member 2	0.71	1.70	0.45*	0
296	gi|4503765	FMR1	fragile X mental retardation protein 1 isoform ISO1	0.76	0.76	0.64	2
297	gi|82546824	FOXK1	forkhead box protein K1	1.24	1.11	1.07	4
298	gi|4507115	FSCN1	fascin	1.30	0.72*	0.59	0
299	gi|194097365	FTSJ3	pre-rRNA processing protein FTSJ3	1.34	0.80	0.27	0
300	gi|17402900	FUBP1	far upstream element-binding protein 1 isoform 2	1.17	1.26	0.31*	3
301	gi|100816392	FUBP3	far upstream element-binding protein 3	1.24	0.65	0.27*	1
302	gi|4826734	FUS	RNA-binding protein FUS isoform 1	1.61	1.25	0.28	0
303	gi|5031703	G3BP1	ras GTPase-activating protein-binding protein 1	0.59	0.57	0.78	0
304	gi|109389365	G6PD	glucose-6-phosphate 1-dehydrogenase isoform a	0.81	0.44*	0.75	0
305	gi|119393891	GAA	lysosomal alpha-glucosidase preproprotein	0.99	1.06	1.56	1
306	gi|4503895	GALK1	galactokinase	1.06	0.82	0.69	0
307	gi|4758412	GALNT2	polypeptide N-acetylgalactosaminyltransferase 2 precursor	1.04	1.16*	2*	0
308	gi|7669492	GAPDH	glyceraldehyde-3-phosphate dehydrogenase isoform 1	1.36*	0.74*	0.30	0
309	gi|116805340	GARS	glycine--tRNA ligase precursor	1.37	0.60	0.52*	0
310	gi|4503915	GART	trifunctional purine biosynthetic protein adenosine-3 isoform 1	0.85	0.58*	0.31*	0
311	gi|54607043	GBA	glucosylceramidase isoform 1 precursor	0.88	1.22	1.5*	0
312	gi|4504011	GCLM	glutamate--cysteine ligase regulatory subunit isoform 1	0.76	1.15	0.23	0

313	gi|54607053	GCN1L1	translational activator GCN1	1.09*	0.75	0.77	1
314	gi|49574537	GCSH	glycine cleavage system H protein, mitochondrial precursor	0.74	1.17	0.81	0
315	gi|4758426	GDA	guanine deaminase isoform b	0.78	0.75*	0.42	0
316	gi|4503971	GDI1	rab GDP dissociation inhibitor alpha	0.99	1.29	0.95	0
317	gi|6598323	GDI2	rab GDP dissociation inhibitor beta isoform 1	0.55	0.59	0.54	0
318	gi|4503987	GGH	gamma-glutamyl hydrolase precursor	0.96	1.77	2.9*	0
319	gi|118402586	GLO1	lactoylglutathione lyase	0.72	1.20	0.13*	0
320	gi|217330598	GLOD4	glyoxalase domain-containing protein 4	1.34	0.79	0.43	0
321	gi|95113651	GLRX3	glutaredoxin-3	0.56	1.05	0.65	0
322	gi|42516576	GLRX5	glutaredoxin-related protein 5, mitochondrial precursor	0.57	0.79	0.61	0
323	gi|4885281	GLUD1	glutamate dehydrogenase 1, mitochondrial precursor	1.07	1.21	1.61	0
324	gi|4504031	GMDS	GDP-mannose 4,6 dehydratase isoform 1	0.74	0.77	0.47	0
325	gi|50541956	GMPR2	GMP reductase 2 isoform 1	0.79	0.61	0.74	0
326	gi|11321585	GNB1	guanine nucleotide-binding protein G(I)/G(S)/G(T) subunit beta-1 isoform 1	0.78	1.10	1.71	0
327	gi|20357529	GNB2	guanine nucleotide-binding protein G(I)/G(S)/G(T) subunit beta-2	0.91	1.09	1.16	0
328	gi|5174447	GNB2L1	guanine nucleotide-binding protein subunit beta-2-like 1	1.33	0.82	0.81	0
329	gi|11055998	GNB4	guanine nucleotide-binding protein subunit beta-4	1.00	1.06	0.75	0
330	gi|51036603	GNG12	guanine nucleotide-binding protein G(I)/G(S)/G(O) subunit gamma-12 precursor	0.75	0.69	0.92	0
331	gi|13027378	GNPDA1	glucosamine-6-phosphate isomerase 1	1.37	1.22	1.33	0
332	gi|4504061	GNS	N-acetylglucosamine-6-sulfatase precursor	0.64	1.01	1.98*	0

333	gi|30089940	GOLGA3	golgin subfamily A member 3 isoform 1	1.86*	1.61*	1.45	1
334	gi|4504067	GOT1	aspartate aminotransferase, cytoplasmic	0.94	0.90	0.3*	0
335	gi|73486658	GOT2	aspartate aminotransferase, mitochondrial isoform 1 precursor	0.72	1.02	1.26	0
336	gi|33356552	GPATCH4	G patch domain-containing protein 4 isoform 1	1.66	1.66*	0.29*	0
337	gi|285002231	GPD2	glycerol-3-phosphate dehydrogenase, mitochondrial precursor	1.02	0.97	0.92	0
338	gi|18201905	GPI	glucose-6-phosphate isomerase isoform 2	0.89	0.89	0.41*	0
339	gi|15811782	GPKOW	G patch domain and KOW motifs-containing protein	0.90	0.77	0.41	0
340	gi|46592991	GRIPAP1	GRIP1-associated protein 1	1.34	0.96	0.86	0
341	gi|24308295	GRPEL1	grpE protein homolog 1, mitochondrial precursor	0.96	1.38	1.38	0
342	gi|149193321	GRSF1	G-rich sequence factor 1 isoform 1	1.00	1.00	0.47	1
343	gi|237820620	GRWD1	glutamate-rich WD repeat-containing protein 1	0.78	0.60	0.33	1
344	gi|194097354	GSPT1	eukaryotic peptide chain release factor GTP-binding subunit ERF3A isoform 2	0.82	0.89	0.65	1
345	gi|4504169	GSS	glutathione synthetase	1.02	1*	0.5*	0
346	gi|23065552	GSTM3	glutathione S-transferase Mu 3	0.50	0.44*	0.94	0
347	gi|254692934	GTF2I	general transcription factor II-I isoform 5	0.76	0.54*	0.65	0
348	gi|55953087	GTPBP4	nucleolar GTP-binding protein 1	1.64*	0.58	0.68	0
349	gi|5174449	H1FX	histone H1x	1.06	1.04	0.39*	0
350	gi|93141018	H2AFY	core histone macro-H2A.1 isoform 3	1.97	1.76	0.81	1
351	gi|20127408	HADHA	trifunctional enzyme subunit alpha, mitochondrial precursor	1.19*	0.57*	0.45*	0
352	gi|4504327	HADHB	trifunctional enzyme subunit beta, mitochondrial isoform 1 precursor	1.02	1.00	0.67	0

353	gi|6996014	HARS	histidine--tRNA ligase, cytoplasmic isoform 1	1.38	1.10	0.47*	0
354	gi|98986457	HCFC1	host cell factor 1	1.11	1.39	1.02	7
355	gi|4758516	HDGF	hepatoma-derived growth factor isoform a	2.12*	1.54	0.48	0
356	gi|4885409	HDLBP	vigilin isoform a	0.89	0.85	0.16*	1
357	gi|73695475	HEATR1	HEAT repeat-containing protein 1	1.95	0.72	1.01	0
358	gi|20336761	HEBP1	heme-binding protein 1	0.86	1.61	0.27*	0
359	gi|4504373	HEXB	beta-hexosaminidase subunit beta isoform 1 preproprotein	1.11	1.18	1.85*	0
360	gi|13124773	HGH1	protein HGH1 homolog	1.46	1.30	0.93	0
361	gi|23308751	HIBADH	3-hydroxyisobutyrate dehydrogenase, mitochondrial precursor	1.03	1.39	1.13	0
362	gi|38045919	HIP1	huntingtin-interacting protein 1 isoform 1	1.00	0.81	1.00	0
363	gi|4885381	HIST1H1B	histone H1.5	0.71	1.75	0.13*	0
364	gi|4885375	HIST1H1C	histone H1.2	1.00	1.84*	0.14*	0
365	gi|4504301	HIST1H4A	histone H4	2.05*	1.31	0.52*	0
366	gi|15553127	HK2	hexokinase-2	1.02	1.30	1.80	0
367	gi|66933009	HMBS	porphobilinogen deaminase isoform 2	1.02	1.02	0.71	0
368	gi|4504425	HMGB1	high mobility group protein B1	1.12	2.08*	0.73	0
369	gi|8051608	HMOX2	heme oxygenase 2 isoform b	2.27	0.93	1.56	0
370	gi|7705877	HN1	hematological and neurological expressed 1 protein isoform 1	0.53	0.82	1.09	0
371	gi|21700763	HN1L	hematological and neurological expressed 1-like protein	0.77	0.89	0.54	0
372	gi|5803036	HNRNPA0	heterogeneous nuclear ribonucleoprotein A0	0.85	0.77	0.33	0

373	gi|14043072	HNRNPA2B1	heterogeneous nuclear ribonucleoproteins A2/B1 isoform B1	1.31*	0.59	0.2*	0
374	gi|34740329	HNRNPA3	heterogeneous nuclear ribonucleoprotein A3	1.22*	0.90	0.35	1
375	gi|55956921	HNRNPAB	heterogeneous nuclear ribonucleoprotein A/B isoform b	1.37*	1.06	0.21*	0
376	gi|332801090	HNRNPDL	heterogeneous nuclear ribonucleoprotein D-like isoform b	0.90	0.83	0.28*	1
377	gi|4826760	HNRNPF	heterogeneous nuclear ribonucleoprotein F	1.35	0.66	0.26*	0
378	gi|5031753	HNRNPH1	heterogeneous nuclear ribonucleoprotein H	1.1*	0.59	0.37	0
379	gi|9624998	HNRNPH2	heterogeneous nuclear ribonucleoprotein H2	1.42	1.25	0.32	0
380	gi|14141159	HNRNPH3	heterogeneous nuclear ribonucleoprotein H3 isoform b	0.96	0.38	0.32	0
381	gi|5031755	HNRNPR	heterogeneous nuclear ribonucleoprotein R isoform 2	0.94	0.81	0.64	5
382	gi|14141161	HNRNPU	heterogeneous nuclear ribonucleoprotein U isoform b	0.89	0.49	0.64	0
383	gi|21536326	HNRNPUL1	heterogeneous nuclear ribonucleoprotein U-like protein 1 isoform a	1.26	0.61	0.23	10
384	gi|4504477	HPD	4-hydroxyphenylpyruvate dioxygenase isoform 1	0.77*	0.84	0.56*	0
385	gi|14249394	HPDL	4-hydroxyphenylpyruvate dioxygenase-like protein	0.70	1.70	0.86	0
386	gi|4504483	HPRT1	hypoxanthine-guanine phosphoribosyltransferase	0.62	0.76	0.35	0
387	gi|5032215	HRSP12	ribonuclease UK114	1.13	1.82	0.95	0
388	gi|4758504	HSD17B10	3-hydroxyacyl-CoA dehydrogenase type-2 isoform 1	1.54	0.79	0.2*	0
389	gi|4504505	HSD17B4	peroxisomal multifunctional enzyme type 2 isoform 2	1.23	0.90	0.96	0
390	gi|40254986	HSDL2	hydroxysteroid dehydrogenase-like protein 2 isoform 1	1.25	0.91	0.46	0
391	gi|431822408	HSP90AB1æ	heat shock protein HSP 90-beta isoform c	1.34*	1.01	0.25*	0
392	gi|4507677	HSP90B1	endoplasmin precursor	1.24*	2.37*	4.15*	0

393	gi|167466173	HSPA1A	heat shock 70 kDa protein 1A/1B	0.76	3.18*	2.37*	0
394	gi|38327039	HSPA4	heat shock 70 kDa protein 4	0.84	0.76*	0.63	0
395	gi|31541941	HSPA4L	heat shock 70 kDa protein 4L	1.01	1.26	0.58*	0
396	gi|16507237	HSPA5	78 kDa glucose-regulated protein precursor	0.91	2.93*	3.5*	1
397	gi|5729877	HSPA8	heat shock cognate 71 kDa protein isoform 1	1.08*	1.39*	0.89	0
398	gi|24234688	HSPA9	stress-70 protein, mitochondrial precursor	0.88*	0.75	0.23*	0
399	gi|4504517	HSPB1	heat shock protein beta-1	0.69	0.47	0.22*	0
400	gi|31542947	HSPD1	60 kDa heat shock protein, mitochondrial	1.13	1.9*	3.24*	0
401	gi|4504523	HSPE1	10 kDa heat shock protein, mitochondrial	0.98	2.39	1.9*	0
402	gi|42544159	HSPH1	heat shock protein 105 kDa isoform 1	0.79	0.59	1.02	0
403	gi|21361437	HTATSF1	HIV Tat-specific factor 1	1.00	0.57*	0.45	0
404	gi|61676188	HUWE1	E3 ubiquitin-protein ligase HUWE1	0.84	0.96	0.89	4
405	gi|5453832	HYOU1	hypoxia up-regulated protein 1 precursor	1.68	1.10	1.63*	1
406	gi|27734984	HYPK	huntingtin-interacting protein K isoform 1	1.13	0.76	0.84	0
407	gi|94721239	IARS	isoleucine--tRNA ligase, cytoplasmic	1.21	0.63	0.5*	0
408	gi|46852147	IARS2	isoleucine--tRNA ligase, mitochondrial precursor	0.92	0.53	0.26*	0
409	gi|167466198	ICAM1	intercellular adhesion molecule 1 precursor	1.17	1.97	2.08*	0
410	gi|29150261	IFI30	gamma-interferon-inducible lysosomal thiol reductase preproprotein	1.77	1.14	1.06	0
411	gi|4557663	IGBP1	immunoglobulin-binding protein 1	0.95	0.88	0.94	0
412	gi|56237027	IGF2BP1	insulin-like growth factor 2 mRNA-binding protein 1 isoform 1	0.98	0.88	0.49	1

413	gi|30795212	IGF2BP3	insulin-like growth factor 2 mRNA-binding protein 3	1.61	0.80	0.30	1
414	gi|119964726	IGF2R	cation-independent mannose-6-phosphate receptor precursor	0.70	0.88	1.40	1
415	gi|55953135	IGSF3	immunoglobulin superfamily member 3 isoform 2 precursor	0.63	1.24	4.83*	0
416	gi|4504653	IL18	interleukin-18 isoform 1 proprotein	0.56	1.27	0.66	0
417	gi|154354966	IMMT	MICOS complex subunit MIC60 isoform 3	1.15	1.24*	1.58*	1
418	gi|34328930	IMPDH1	inosine-5'-monophosphate dehydrogenase 1 isoform a	1.24	0.32*	0.93*	1
419	gi|66933016	IMPDH2	inosine-5'-monophosphate dehydrogenase 2	1.20	0.86	0.3*	0
420	gi|222136583	INPPL1	phosphatidylinositol 3,4,5-trisphosphate 5-phosphatase 2	0.80	1.17	1.87	7
421	gi|24797086	IPO5	importin-5	0.60	0.69*	0.96	0
422	gi|5453998	IPO7	importin-7	0.75	0.71	2.49	0
423	gi|4506787	IQGAP1	ras GTPase-activating-like protein IQGAP1	0.76*	0.59*	0.68	0
424	gi|103471987	ISOC1	isochorismatase domain-containing protein 1	0.77	0.82	0.31	0
425	gi|19743813	ITGB1	integrin beta-1 isoform 1A precursor	1.30	1.24*	2.74*	0
426	gi|5031815	KARS	lysine--tRNA ligase isoform 2	1.45	2.77*	0.37*	1
427	gi|5730027	KHDRBS1	KH domain-containing, RNA-binding, signal transduction-associated protein 1 isoform 1	1.39*	0.64*	0.57*	4
428	gi|154355000	KHSRP	far upstream element-binding protein 2	1.02	1.02	0.34*	7
429	gi|122937211	KIAA0368	proteasome-associated protein ECM29 homolog	0.81	0.93	0.49	2
430	gi|4758648	KIF5B	kinesin-1 heavy chain	1.37	0.57	0.56	0
431	gi|4504897	KPNA2	importin subunit alpha-1	0.59	0.47*	1.49	0
432	gi|4504901	KPNA4	importin subunit alpha-3	0.86	0.93	1.23	1

433	gi|19923142	KPNB1	importin subunit beta-1 isoform 1	0.71	1.01*	2.14*	0
434	gi|4557701	KRT17	keratin, type I cytoskeletal 17	0.99*	1.34*	1.47	0
435	gi|4557888	KRT18	keratin, type I cytoskeletal 18	0.95*	0.77	1.56*	0
436	gi|47132620	KRT2	keratin, type II cytoskeletal 2 epidermal	1.02	0.86	0.56	0
437	gi|67782365	KRT7	keratin, type II cytoskeletal 7	0.77*	0.62	0.98	0
438	gi|4504919	KRT8	keratin, type II cytoskeletal 8 isoform 2	1.05*	0.75	1.64*	0
439	gi|55956899	KRT9	keratin, type I cytoskeletal 9	0.37*	1.37*	1.30	0
440	gi|221316760	L1CAM	neural cell adhesion molecule L1 isoform 3 precursor	0.91	0.86	0.90	1
441	gi|167614504	LAMB1	laminin subunit beta-1 precursor	0.85	1.49	2.02*	0
442	gi|119703755	LAMB2	laminin subunit beta-2 precursor	0.72*	1.14	0.35*	0
443	gi|112380628	LAMP1	lysosome-associated membrane glycoprotein 1 precursor	0.69	1.27	3.36*	0
444	gi|5174445	LANCL1	lanC-like protein 1	0.40	0.41	0.33	0
445	gi|41393561	LAP3	cytosol aminopeptidase	1.67	1.21	0.44*	0
446	gi|108773810	LARS	leucine--tRNA ligase, cytoplasmic	1.50	0.71	0.5*	0
447	gi|5453710	LASP1	LIM and SH3 domain protein 1 isoform a	1.33	1.21	0.22*	0
448	gi|4557032	LDHB	L-lactate dehydrogenase B chain	1.06	0.86	0.2*	0
449	gi|4504975	LDLR	low-density lipoprotein receptor isoform 1 precursor	0.74	1.36	3.47*	0
450	gi|31044432	LEMD2	LEM domain-containing protein 2 isoform 1	1.06	0.62	1.65	0
451	gi|6912482	LETM1	LETM1 and EF-hand domain-containing protein 1, mitochondrial precursor	0.57	1.07	3.91*	1
452	gi|4504981	LGALS1	galectin-1	1.57	1.23	0.88	0

453	gi|5031863	LGALS3BP	galectin-3-binding protein precursor	1.08	1.25	1.82*	0
454	gi|7705373	LIMA1	LIM domain and actin-binding protein 1 isoform 2	0.92	1.00	0.59*	1
455	gi|7657307	LIMD1	LIM domain-containing protein 1	0.96	1.04	1.15	0
456	gi|27436946	LMNA	lamin isoform A	1.82*	1.44*	1.54*	0
457	gi|5031877	LMNB1	lamin-B1 isoform 1	1.28	1.27*	1.41*	0
458	gi|388240801	LMNB2	lamin-B2	1.11	1.31	3.33*	0
459	gi|33598968	LMO7	LIM domain only protein 7 isoform 1	0.94	1.23	0.64	2
460	gi|21396489	LONP1	lon protease homolog, mitochondrial isoform 1 precursor	0.92	0.52*	0.19*	0
461	gi|31621305	LRPPRC	leucine-rich PPR motif-containing protein, mitochondrial precursor	0.68	0.66	0.62	0
462	gi|40254924	LRRC59	leucine-rich repeat-containing protein 59	0.38	2.18	5.86*	1
463	gi|7657315	LSM3	U6 snRNA-associated Sm-like protein LSm3	0.95	1.57	0.86	0
464	gi|4505029	LTA4H	leukotriene A-4 hydrolase isoform 1	1.46	1.28	0.53*	0
465	gi|19923485	LUC7L3	luc7-like protein 3	0.96	0.56	0.76	0
466	gi|4505061	M6PR	cation-dependent mannose-6-phosphate receptor isoform 1 precursor	0.68	0.91	3.19	0
467	gi|33188445	MACF1	microtubule-actin cross-linking factor 1	0.85	0.97	0.98	2
468	gi|19387846	MAGED2	melanoma-associated antigen D2	1.40	0.57	1.13	1
469	gi|51873064	MAN2B1	lysosomal alpha-mannosidase isoform 1 precursor	0.78	0.98	1.43	0
470	gi|197276600	MAP4	microtubule-associated protein 4 isoform 4	1.19	1.18	0.25*	1
471	gi|153070260	MARCKS	myristoylated alanine-rich C-kinase substrate	1.05	1.67*	1.02	0
472	gi|14043022	MARS	methionine--tRNA ligase, cytoplasmic	1.08	0.52	0.98*	1

473	gi|5174529	MAT2A	S-adenosylmethionine synthase isoform type-2	0.96	0.89	0.4*	0
474	gi|21626466	MATR3	matrin-3 isoform a	0.76	0.29	0.60	1
475	gi|71274107	MCAM	cell surface glycoprotein MUC18 precursor	1.35	0.67	2.04	1
476	gi|33356547	MCM2	DNA replication licensing factor MCM2	0.63	0.47*	0.48	0
477	gi|33469917	MCM4	DNA replication licensing factor MCM4	1.01	0.62	0.70	0
478	gi|23510448	MCM5	DNA replication licensing factor MCM5	1.53	0.59*	0.4*	0
479	gi|7427519	MCM6	DNA replication licensing factor MCM6	0.85	0.44	0.59	0
480	gi|33469968	MCM7	DNA replication licensing factor MCM7 isoform 1	1.02*	0.72	0.36*	0
481	gi|5174539	MDH1	malate dehydrogenase, cytoplasmic isoform 2	0.89	0.70	0.3*	0
482	gi|21735621	MDH2	malate dehydrogenase, mitochondrial isoform 1 precursor	1.40	1.89*	1.36*	0
483	gi|4505145	ME2	NAD-dependent malic enzyme, mitochondrial isoform 1 precursor	1.81	3.68*	1.85	0
484	gi|74136552	MESDC2	LDLR chaperone MESD precursor	1.07	1.86	1.97*	2
485	gi|5803092	METAP2	methionine aminopeptidase 2	1.55	1.30	0.35*	0
486	gi|122891870	MIA3	melanoma inhibitory activity protein 3 isoform 1 precursor	1.28	1.13	2.07*	7
487	gi|4505185	MIF	macrophage migration inhibitory factor	0.72	2.10	0.14	0
488	gi|27735067	MISP	mitotic interactor and substrate of PLK1	1.05	1.26	0.52	0
489	gi|31317305	MPHOSPH10	U3 small nucleolar ribonucleoprotein protein MPP10	2.45	0.71	0.83	0
490	gi|61835204	MPST	3-mercaptopyruvate sulfurtransferase isoform 2	1.56*	1.23	0.56	0
491	gi|27436901	MRPL12	39S ribosomal protein L12, mitochondrial	0.55	0.98	1.45	0
492	gi|26638659	MRPL40	39S ribosomal protein L40, mitochondrial	1.20	0.53	0.61	0

493	gi|12597661	MRPL44	39S ribosomal protein L44, mitochondrial	2.61	0.77	2.66	1
494	gi|186928854	MRPS31	28S ribosomal protein S31, mitochondrial	0.69	0.72	1.09	0
495	gi|18490987	MRTO4	mRNA turnover protein 4 homolog	0.85	0.91	1.20	0
496	gi|4557761	MSH2	DNA mismatch repair protein Msh2 isoform 1	0.81	0.76*	0.43	0
497	gi|4505257	MSN	moesin	1.19	0.98	0.38*	0
498	gi|15011880	MST4	serine/threonine-protein kinase 26 isoform 1	1.53	1.11	0.24	0
499	gi|14141170	MTA2	metastasis-associated protein MTA2	1.05	0.64	1.03	0
500	gi|47132622	MTAP	S-methyl-5'-thioadenosine phosphorylase	0.72	2.03	0.30	0
501	gi|222136639	MTHFD1	C-1-tetrahydrofolate synthase, cytoplasmic	1.26	0.70	0.26*	0
502	gi|36796743	MTHFD1L	monofunctional C1-tetrahydrofolate synthase, mitochondrial isoform 2 precursor	0.85	0.45*	0.36*	0
503	gi|169790956	MTRR	methionine synthase reductase isoform 2	1.12	0.65	1.31	0
504	gi|19913410	MVP	major vault protein isoform 1	0.58	1.07	2.84*	0
505	gi|12667788	MYH9	myosin-9	1.16	1.47*	2.63*	0
506	gi|17149828	NAA15	N-alpha-acetyltransferase 15, NatA auxiliary subunit	1.29	0.91	1.13	0
507	gi|333033787	NACA	nascent polypeptide-associated complex subunit alpha isoform a	0.74	1.16	0.39	1
508	gi|5031977	NAMPT	nicotinamide phosphoribosyltransferase precursor	1.15	0.64*	0.53	0
509	gi|12056473	NANS	sialic acid synthase	1.34	0.41	0.32	0
510	gi|4758756	NAP1L1	nucleosome assembly protein 1-like 1 isoform 1	0.84	1.02	2.51*	0
511	gi|5174613	NAP1L4	nucleosome assembly protein 1-like 4	0.66	1.55	0.81	0
512	gi|194394158	NAPRT1	nicotinate phosphoribosyltransferase isoform 1	0.81	0.62	0.22*	0

513	gi|4758762	NARS	asparagine--tRNA ligase, cytoplasmic	0.85	0.72*	0.41*	0
514	gi|27262628	NASP	nuclear autoantigenic sperm protein isoform 2	0.77	1.22	0.68	0
515	gi|68051721	NCEH1	neutral cholesterol ester hydrolase 1 isoform b	1.11	1.18	1.33	0
516	gi|55956788	NCL	nucleolin	1.40	1.45	0.28*	1
517	gi|24638433	NCSTN	nicastrin isoform 1 precursor	1.47	1.12	2.12	0
518	gi|37655183	NDRG1	protein NDRG1 isoform 1	0.80	0.87	1.12	0
519	gi|7019545	NENF	neudesin precursor	1.03	1.95*	2.9*	0
520	gi|38176300	NES	nestin	0.77	1.06	1.51	1
521	gi|117320531	NFKB2	nuclear factor NF-kappa-B p100 subunit isoform a	1.00	0.99	0.68	1
522	gi|42476013	NHLRC2	NHL repeat-containing protein 2	1.18	0.71*	0.57	0
523	gi|4826860	NHP2L1	NHP2-like protein 1	1.58	1.14	1.08	0
524	gi|222352111	NIFK	MKI67 FHA domain-interacting nucleolar phosphoprotein	0.81	0.63	0.52	0
525	gi|9910460	NIT2	omega-amidase NIT2	1.43	2.00	0.50	0
526	gi|10835073	NMT1	glycylpeptide N-tetradecanoyltransferase 1	1*	0.66*	0.80	0
527	gi|5453790	NNMT	nicotinamide N-methyltransferase	0.73	0.58	0.30	0
528	gi|7661532	NOB1	RNA-binding protein NOB1	1.04	0.51*	0.44	0
529	gi|148596949	NOLC1	nucleolar and coiled-body phosphoprotein 1 isoform 2	0.87	0.70	0.48*	3
530	gi|34932414	NONO	non-POU domain-containing octamer-binding protein isoform 1	0.85	0.92	0.96	1
531	gi|148747209	NOP16	nucleolar protein 16 isoform 3	0.98	0.91	0.55	0
532	gi|32483374	NOP56	nucleolar protein 56	1.13	0.89	0.34	0

533	gi|24041035	NOTCH2	neurogenic locus notch homolog protein 2 isoform 1 preproprotein	0.68	1.18	8.87	0
534	gi|158937236	NPEPPS	puromycin-sensitive aminopeptidase	0.95	0.5*	0.36*	1
535	gi|157426879	NPLOC4	nuclear protein localization protein 4 homolog	2.16	1.12	0.34	0
536	gi|10835063	NPM1	nucleophosmin isoform 1	0.73	1.05	0.95	0
537	gi|6857818	NPM3	nucleoplasmin-3	1.21	1.04	1.00	0
538	gi|156564401	NSF	vesicle-fusing ATPase	0.82	0.58	0.73	0
539	gi|332078466	NSFL1C	NSFL1 cofactor p47 isoform d	1.04	0.99	0.80	0
540	gi|39995082	NSUN2	tRNA (cytosine(34)-C(5))-methyltransferase isoform 1	1.08	0.68*	0.4*	1
541	gi|38570156	NT5DC1	5'-nucleotidase domain-containing protein 1	1.85	0.77	0.71	0
542	gi|6912540	NUBP2	cytosolic Fe-S cluster assembly factor NUBP2 isoform 1	0.79	0.24	0.86	0
543	gi|20070228	NUCB1	nucleobindin-1 precursor	0.49	0.86	1.64	0
544	gi|5729953	NUDC	nuclear migration protein nudC	0.70	0.91	0.41	0
545	gi|21687129	NUDCD2	nudC domain-containing protein 2	1.05	1.21	0.69	0
546	gi|37594464	NUDT5	ADP-sugar pyrophosphatase	0.76	1.31	0.51*	0
547	gi|71361682	NUMA1	nuclear mitotic apparatus protein 1 isoform 1	0.85	1.19	1.05	0
548	gi|24430149	NUP155	nuclear pore complex protein Nup155 isoform 1	0.64	0.72	1.34	1
549	gi|27477134	NUP210	nuclear pore membrane glycoprotein 210 precursor	1.03	0.81	1.58*	0
550	gi|33946327	NUP214	nuclear pore complex protein Nup214	1.57	2.12	4.27	1
551	gi|38605733	NUP43	nucleoporin Nup43	1.10	1.22	1.65	0
552	gi|24497451	NUP50	nuclear pore complex protein Nup50 isoform b	1.01	0.75	0.97	0

553	gi|208609990	NUP93	nuclear pore complex protein Nup93 isoform 1	1.10	0.78	2.52*	0
554	gi|5031985	NUTF2	nuclear transport factor 2	0.75	0.71	0.20	0
555	gi|15487670	NXF1	nuclear RNA export factor 1 isoform 1	0.98	0.73	1.12	1
556	gi|33149331	NXN	nucleoredoxin isoform 1	1.45	0.87	0.88	0
557	gi|4557809	OAT	ornithine aminotransferase, mitochondrial isoform 1 precursor	0.59	0.50	0.89	0
558	gi|51873036	OGDH	2-oxoglutarate dehydrogenase, mitochondrial isoform 1 precursor	1.20	1.13	1.96	0
559	gi|58761500	OLA1	obg-like ATPase 1 isoform 1	0.89	1.03	0.67*	1
560	gi|4557817	OXCT1	succinyl-CoA:3-ketoacid coenzyme A transferase 1, mitochondrial precursor	1.42	0.75	0.86	0
561	gi|4826878	OXSR1	serine/threonine-protein kinase OSR1	1.58*	0.94	0.39	0
562	gi|20070125	P4HB	protein disulfide-isomerase precursor	0.88*	2.81*	3.39*	0
563	gi|124494254	PA2G4	proliferation-associated protein 2G4	1.12*	1.11*	0.35*	0
564	gi|46367787	PABPC1	polyadenylate-binding protein 1	1.19	0.61	1.27	0
565	gi|19224660	PACSIN3	protein kinase C and casein kinase substrate in neurons protein 3	0.73	0.87	1.14	1
566	gi|5453539	PAICS	multifunctional protein ADE2 isoform 2	0.73	0.89	0.46*	0
567	gi|31543380	PARK7	protein DJ-1	1.15	0.94	0.28	0
568	gi|19923830	PBXIP1	pre-B-cell leukemia transcription factor-interacting protein 1	0.92	1.10	1.73	0
569	gi|106049292	PC	pyruvate carboxylase, mitochondrial precursor	0.82	0.55	0.19*	1
570	gi|222352151	PCBP1	poly(rC)-binding protein 1	0.92	0.75	0.40	0
571	gi|354983493	PCMT1	protein-L-isoaspartate(D-aspartate) O-methyltransferase isoform 2	1.97	1.01	0.4*	0
572	gi|4505641	PCNA	proliferating cell nuclear antigen	0.89	1.26*	0.49*	0

573	gi|9966827	PCNP	PEST proteolytic signal-containing nuclear protein	1.06	1.23	0.16*	0
574	gi|166795301	PCYOX1	prenylcysteine oxidase 1 precursor	1.21	1.73	1.75	0
575	gi|7657441	PDAP1	28 kDa heat- and acid-stable phosphoprotein	1.00	0.91	0.48	0
576	gi|70980549	PDCD11	protein RRP5 homolog	0.84	0.63	0.63	0
577	gi|313760537	PDCD4	programmed cell death protein 4 isoform 3	0.97*	0.33*	0.15*	0
578	gi|4759224	PDCD5	programmed cell death protein 5	0.86	0.83	0.27*	0
579	gi|7019485	PDCD6	programmed cell death protein 6 isoform 1	0.64	0.63	3.5*	0
580	gi|189027129	PDE12	2',5'-phosphodiesterase 12	0.87	0.90	0.81	0
581	gi|21361657	PDIA3	protein disulfide-isomerase A3 precursor	1.07	1.56*	3.34*	0
582	gi|4758304	PDIA4	protein disulfide-isomerase A4 precursor	1.07*	2.48	2.72*	1
583	gi|5031973	PDIA6	protein disulfide-isomerase A6 isoform d precursor	1.15	1.51	2.14*	1
584	gi|13994151	PDLIM1	PDZ and LIM domain protein 1	1.11	0.68*	0.43	0
585	gi|190341074	PDXDC1	pyridoxal-dependent decarboxylase domain-containing protein 1 isoform 1	0.71	0.66	0.47	0
586	gi|4505701	PDXK	pyridoxal kinase	1.5*	1.08	0.57	0
587	gi|4505621	PEBP1	phosphatidylethanolamine-binding protein 1	0.82*	0.87	0.3*	0
588	gi|31657129	PFAS	phosphoribosylformylglycinamidine synthase	1.00	0.96	0.88	1
589	gi|22202633	PFDN5	prefoldin subunit 5 isoform alpha	1.06	0.89	0.78	0
590	gi|7657162	PFDN6	prefoldin subunit 6	1.94	1.74	0.33*	0
591	gi|48762920	PFKL	ATP-dependent 6-phosphofructokinase, liver type isoform b	0.64	0.96	1.09	0
592	gi|4826898	PFN1	profilin-1	1.00	0.71	0.34*	0

593	gi|4505753	PGAM1	phosphoglycerate mutase 1	1.08	0.99	0.19*	1
594	gi|281604138	PGAM5	serine/threonine-protein phosphatase PGAM5, mitochondrial isoform 2	0.87	1.05	0.74	0
595	gi|40068518	PGD	6-phosphogluconate dehydrogenase, decarboxylating isoform 1	0.75	0.47	0.50	0
596	gi|4505763	PGK1	phosphoglycerate kinase 1	1.05	0.89	0.22*	0
597	gi|6912586	PGLS	6-phosphogluconolactonase	1.22	0.99	0.27*	1
598	gi|21361621	PGM1	phosphoglucomutase-1 isoform 1	0.98	0.82	0.24*	0
599	gi|108796653	PGP	phosphoglycolate phosphatase	1.01	0.82	0.47	0
600	gi|5729875	PGRMC1	membrane-associated progesterone receptor component 1 isoform 1	0.75	1.75	2.72*	1
601	gi|4505773	PHB	prohibitin isoform 1	1.12*	1.2*	2.48*	0
602	gi|221307584	PHB2	prohibitin-2 isoform 1	0.96	1.15	1.25*	0
603	gi|23308577	PHGDH	D-3-phosphoglycerate dehydrogenase	1.27*	0.72*	0.38*	0
604	gi|4505823	PIR	pirin	0.79	1.29	0.42	0
605	gi|33286418	PKM-a	pyruvate kinase PKM isoform a	1.12	0.98	0.58*	0
606	gi|332164775	PKM-c	pyruvate kinase PKM isoform c	1.00	1.06*	0.49	0
607	gi|5453974	PKN2	serine/threonine-protein kinase N2	0.89	0.80	0.26*	1
608	gi|32307144	PLOD1	procollagen-lysine,2-oxoglutarate 5-dioxygenase 1 precursor	1.07	2.12*	2.25*	0
609	gi|4505891	PLOD3	procollagen-lysine,2-oxoglutarate 5-dioxygenase 3 precursor	0.93	1.50	3.3*	1
610	gi|7549809	PLS3	plastin-3 isoform 1	0.84	0.74	0.37*	0
611	gi|4557839	PMM2	phosphomannomutase 2	0.9*	0.75*	0.58	0
612	gi|24308013	PMPCA	mitochondrial-processing peptidase subunit alpha isoform 1 precursor	1.75	0.57	1.13	0

613	gi|94538354	PMPCB	mitochondrial-processing peptidase subunit beta precursor	0.70	0.72	0.75	0
614	gi|157168362	PNP	purine nucleoside phosphorylase	0.91	1.27	0.53	0
615	gi|4505941	POLR2B	DNA-directed RNA polymerase II subunit RPB2 isoform 1	1.07	0.82	0.87	0
616	gi|127139033	POR	NADPH--cytochrome P450 reductase	0.81	0.57	1.13	0
617	gi|11056044	PPA1	inorganic pyrophosphatase	1.92	0.93	0.35	1
618	gi|29570798	PPAT	amidophosphoribosyltransferase proprotein	0.72	1.07	0.76	0
619	gi|10863927	PPIA	peptidyl-prolyl cis-trans isomerase A isoform 1	0.96	0.65*	0.19*	0
620	gi|4758950	PPIB	peptidyl-prolyl cis-trans isomerase B precursor	0.79	0.95*	2.72*	0
621	gi|5174637	PPIE	peptidyl-prolyl cis-trans isomerase E isoform 1	0.72	0.81	1.91	0
622	gi|29826282	PPM1G	protein phosphatase 1G	1.13	1.73	0.53	0
623	gi|145386517	PPP1R18	phostensin	0.98	1.16	0.65	2
624	gi|4506017	PPP2CA	serine/threonine-protein phosphatase 2A catalytic subunit alpha isoform	1.45*	0.67	0.22*	0
625	gi|21361399	PPP2R1A	serine/threonine-protein phosphatase 2A 65 kDa regulatory subunit A alpha isoform	1.19	0.58	1.00	0
626	gi|4506027	PPP4C	serine/threonine-protein phosphatase 4 catalytic subunit isoform 1	0.73	0.67	0.81	0
627	gi|4826934	PPP4R1	serine/threonine-protein phosphatase 4 regulatory subunit 1 isoform b	0.64	0.58*	1.25*	0
628	gi|5453958	PPP5C	serine/threonine-protein phosphatase 5 isoform 1	1.03	1.35	0.48	0
629	gi|4505591	PRDX1	peroxiredoxin-1	0.78	1.02	0.22*	0
630	gi|32189392	PRDX2	peroxiredoxin-2	1.19	0.90	0.29*	0
631	gi|5802974	PRDX3	thioredoxin-dependent peroxide reductase, mitochondrial isoform a precursor	0.33	0.79*	1.20	0
632	gi|5453549	PRDX4	peroxiredoxin-4 precursor	0.95	1.15	2.1*	0

633	gi|6912238	PRDX5	peroxiredoxin-5, mitochondrial isoform a precursor	1.15	1.06	0.37*	0
634	gi|4758638	PRDX6	peroxiredoxin-6	0.83*	0.74	0.24*	0
635	gi|41349456	PREP	prolyl endopeptidase	0.81	0.96	0.69	0
636	gi|4506063	PRKAR1A	cAMP-dependent protein kinase type I-alpha regulatory subunit isoform a	1.65	0.36	0.36	1
637	gi|4758958	PRKAR2A	cAMP-dependent protein kinase type II-alpha regulatory subunit	1.21	1.02	0.52	1
638	gi|48255891	PRKCSH	glucosidase 2 subunit beta isoform 2 precursor	1.46	2.37*	2.4*	1
639	gi|20070220	PRMT5	protein arginine N-methyltransferase 5 isoform a	0.96	0.92	0.54	1
640	gi|7657381	PRPF19	pre-mRNA-processing factor 19	0.74	1.05	1.15	0
641	gi|4758556	PRPF3	U4/U6 small nuclear ribonucleoprotein Prp3	0.73	0.98	0.79	1
642	gi|91208426	PRPF8	pre-mRNA-processing-splicing factor 8	1.12	0.68	2.14	1
643	gi|84875539	PRPS2	ribose-phosphate pyrophosphokinase 2 isoform 1	0.90	0.77	0.60	0
644	gi|194018537	PRPSAP1	phosphoribosyl pyrophosphate synthase-associated protein 1	1.04	0.95	0.66*	2
645	gi|4506133	PRPSAP2	phosphoribosyl pyrophosphate synthase-associated protein 2 isoform 1	0.71	0.71	0.91	0
646	gi|11386147	PSAP	prosaposin isoform a preproprotein	0.77	1.25	1.82*	0
647	gi|17402893	PSAT1	phosphoserine aminotransferase isoform 1	0.67	0.58	0.63*	0
648	gi|19923653	PSIP1	PC4 and SFRS1-interacting protein isoform 2	0.82	0.93	0.68	0
649	gi|4506181	PSMA2	proteasome subunit alpha type-2	0.95	0.93*	0.32	0
650	gi|4506183	PSMA3	proteasome subunit alpha type-3 isoform 1	0.68	1.11	0.39	0
651	gi|4506185	PSMA4	proteasome subunit alpha type-4 isoform 1	0.40	0.81	0.31	0
652	gi|23110942	PSMA5	proteasome subunit alpha type-5 isoform 1	1.05	0.92	0.37	0

653	gi|23110944	PSMA6	proteasome subunit alpha type-6 isoform a	1.10	0.76	0.39*	0
654	gi|4506189	PSMA7	proteasome subunit alpha type-7	0.91	0.84	0.68	0
655	gi|4506193	PSMB1	proteasome subunit beta type-1	0.98	1.08	0.78	0
656	gi|4506195	PSMB2	proteasome subunit beta type-2 isoform 1	1.00	1.12	0.78	0
657	gi|22538465	PSMB3	proteasome subunit beta type-3	0.57	1.08	0.52	0
658	gi|22538467	PSMB4	proteasome subunit beta type-4	1.18	0.93	0.53*	0
659	gi|4506201	PSMB5	proteasome subunit beta type-5 isoform 1	1.13	0.97	0.94	0
660	gi|23110925	PSMB6	proteasome subunit beta type-6 isoform 1 proprotein	0.73	0.77	0.82	0
661	gi|4506203	PSMB7	proteasome subunit beta type-7 proprotein	0.71	0.94	0.61	0
662	gi|24430151	PSMC1	26S protease regulatory subunit 4	0.96	0.52	0.71	0
663	gi|4506209	PSMC2	26S protease regulatory subunit 7 isoform 1	1.20	0.74	0.64	0
664	gi|21361144	PSMC3	26S protease regulatory subunit 6A	0.96	0.86	0.80	0
665	gi|5729991	PSMC4	26S protease regulatory subunit 6B isoform 1	1.09	0.39*	0.58	0
666	gi|312596881	PSMC5	26S protease regulatory subunit 8 isoform 2	1.04	0.88	0.74	1
667	gi|195539395	PSMC6	26S protease regulatory subunit 10B	1.18	1.09	0.74	0
668	gi|4506221	PSMD12	26S proteasome non-ATPase regulatory subunit 12 isoform 1	0.49	0.41	2.83	0
669	gi|25777602	PSMD2	26S proteasome non-ATPase regulatory subunit 2 isoform 1	1.17	0.66	1.27	0
670	gi|25777612	PSMD3	26S proteasome non-ATPase regulatory subunit 3	0.71	0.59	1.54	2
671	gi|4826952	PSMD5	26S proteasome non-ATPase regulatory subunit 5 isoform 1	0.56	1.11	1.08	0
672	gi|7661914	PSMD6	26S proteasome non-ATPase regulatory subunit 6 isoform 2	0.71	0.44	1.76	0

673	gi|25777615	PSMD7	26S proteasome non-ATPase regulatory subunit 7	0.71	0.85	0.94	0
674	gi|156631005	PSMD8	26S proteasome non-ATPase regulatory subunit 8	0.73	1.36	4.25*	0
675	gi|18543329	PSMD9	26S proteasome non-ATPase regulatory subunit 9 isoform 1	0.87	0.93	0.67	0
676	gi|5453990	PSME1	proteasome activator complex subunit 1 isoform 1	1.07	0.79	0.81	0
677	gi|30410796	PSME3	proteasome activator complex subunit 3 isoform 2	1.00	1.05	4.53*	0
678	gi|46249388	PSPH	phosphoserine phosphatase	1.26	1.08	0.66	0
679	gi|23308579	PTGES3	prostaglandin E synthase 3 isoform a	0.79	0.86	0.2*	0
680	gi|42734430	PTRF	polymerase I and transcript release factor	1.09	0.76	0.59	0
681	gi|5902034	PWP1	periodic tryptophan protein 1 homolog	0.71	1.08	1.51	0
682	gi|24797097	PYCR1	pyrroline-5-carboxylate reductase 1, mitochondrial isoform 1	0.99	2.56	1.03	0
683	gi|4826960	QARS	glutamine--tRNA ligase isoform a	0.91	0.56*	0.93	0
684	gi|13654276	QTRT1	queuine tRNA-ribosyltransferase	0.79	0.99	0.51	0
685	gi|256222019	RAB10	ras-related protein Rab-10	1.52	0.97	0.97	0
686	gi|4758988	RAB1A	ras-related protein Rab-1A isoform 1	0.92	1.07	1.02	0
687	gi|13569962	RAB1B	ras-related protein Rab-1B	1.23	1.83	0.67	0
688	gi|19923262	RAB5A	ras-related protein Rab-5A isoform 1	1.12	1.47	1.61	0
689	gi|4506371	RAB5B	ras-related protein Rab-5B isoform 1	1.06	0.79	0.78	0
690	gi|34147513	RAB7A	ras-related protein Rab-7a	0.94	0.84	0.89	0
691	gi|16933567	RAB8A	ras-related protein Rab-8A	0.84	0.73	0.37*	0
692	gi|31377798	RABEP1	rab GTPase-binding effector protein 1 isoform 1	1.35	0.86	2.47	0

693	gi|4506387	RAD23B	UV excision repair protein RAD23 homolog B isoform 1	0.32	1.23	0.97	0
694	gi|19924129	RAD50	DNA repair protein RAD50	0.88	1.23	0.79	0
695	gi|5453555	RAN	GTP-binding nuclear protein Ran isoform 1	0.93*	0.49	0.26	0
696	gi|4506407	RANBP1	ran-specific GTPase-activating protein isoform 2	0.79	0.79	0.31	0
697	gi|150418007	RANBP2	E3 SUMO-protein ligase RanBP2	0.86	0.89	0.85*	1
698	gi|4506411	RANGAP1	ran GTPase-activating protein 1	1.12	1.54	0.52	1
699	gi|15149476	RARS	arginine--tRNA ligase, cytoplasmic	0.99	0.53	1.58	0
700	gi|5032027	RBBP4	histone-binding protein RBBP4 isoform a	1.38	1.36	1.49	0
701	gi|19923345	RBM12	RNA-binding protein 12	0.89	1.03	0.94	6
702	gi|5454064	RBM14	RNA-binding protein 14 isoform 1	1.23	0.65*	0.57	1
703	gi|55741709	RBM25	RNA-binding protein 25	0.77	0.67	1.07	2
704	gi|31652264	RBM26	RNA-binding protein 26 isoform 3	0.70	0.51	0.37	7
705	gi|4757926	RBM39	RNA-binding protein 39 isoform b	1.54	0.61	0.48	0
706	gi|4826972	RBM8A	RNA-binding protein 8A	0.88	1.04	0.78	0
707	gi|29789090	RCC2	protein RCC2	1.27	0.77	0.39*	0
708	gi|4506455	RCN1	reticulocalbin-1 precursor	2.17*	1.58	1.68	0
709	gi|28626510	RCN3	reticulocalbin-3 precursor	1.16	1.45*	1.89	0
710	gi|4506491	RFC4	replication factor C subunit 4	1.14	1.24	0.58	0
711	gi|5032041	RHEB	GTP-binding protein Rheb	1.24	0.58	0.66	0
712	gi|10835049	RHOA	transforming protein RhoA precursor	0.79	1.30	0.78	0

713	gi|21361547	RNH1	ribonuclease inhibitor	1.11	1.20	0.27	0
714	gi|40316915	RNPEP	aminopeptidase B	1.15	0.89	0.42*	0
715	gi|41872583	ROCK2	rho-associated protein kinase 2	0.71	0.91	0.68	2
716	gi|223890243	RPL10	60S ribosomal protein L10 isoform a	0.96	1.46*	0.4*	0
717	gi|15431288	RPL10A	60S ribosomal protein L10a	1.88	2.21	0.87	0
718	gi|4506597	RPL12	60S ribosomal protein L12	1.30	1.08	0.71	0
719	gi|6912634	RPL13A	60S ribosomal protein L13a isoform 1	0.99	1.01	0.29*	1
720	gi|78000181	RPL14	60S ribosomal protein L14	1.60	1.38	0.28*	0
721	gi|4506617	RPL17	60S ribosomal protein L17 isoform a	1.60	1.18	0.43	0
722	gi|4506607	RPL18	60S ribosomal protein L18 isoform 1	0.89*	1.16	0.39	0
723	gi|4506609	RPL19	60S ribosomal protein L19	1.07	1.21*	0.17*	0
724	gi|4506613	RPL22	60S ribosomal protein L22 proprotein	0.87	1.19	0.48	0
725	gi|4506605	RPL23	60S ribosomal protein L23	0.78	0.79	0.44	0
726	gi|17105394	RPL23A	60S ribosomal protein L23a	1.21	1.26	0.61*	0
727	gi|4506619	RPL24	60S ribosomal protein L24	1.31*	1.72	0.25*	0
728	gi|4506625	RPL27A	60S ribosomal protein L27a	1.01	1.11	0.56	0
729	gi|16117787	RPL34	60S ribosomal protein L34	1.35	1.08	0.35	0
730	gi|6005860	RPL35	60S ribosomal protein L35	2.06	1.74	0.19	0
731	gi|16579885	RPL4	60S ribosomal protein L4	1.19	1.71*	0.27*	0
732	gi|16753227	RPL6	60S ribosomal protein L6	2.08	1.41	0.26*	0

733	gi|15431301	RPL7	60S ribosomal protein L7	1.46*	1.15	0.29	0
734	gi|4506661	RPL7A	60S ribosomal protein L7a	1.00	1.49	0.44*	0
735	gi|4506663	RPL8	60S ribosomal protein L8	1.33	1.15	0.29*	0
736	gi|15431303	RPL9	60S ribosomal protein L9	1.39	0.58	0.52	0
737	gi|4506667	RPLP0	60S acidic ribosomal protein P0	1.09*	0.87	2.20	0
738	gi|4506669	RPLP1	60S acidic ribosomal protein P1 isoform 1	1.16*	1.79*	2.75*	0
739	gi|4506671	RPLP2	60S acidic ribosomal protein P2	0.66	0.81	2.11*	0
740	gi|4506675	RPN1	dolichyl-diphosphooligosaccharide--protein glycosyltransferase subunit 1 precursor	1.29*	2.21*	2.48*	0
741	gi|183396804	RPRD2	regulation of nuclear pre-mRNA domain-containing protein 2 isoform 1	1.36*	0.95*	0.74	7
742	gi|4506679	RPS10	40S ribosomal protein S10	2.53	0.64	0.28	0
743	gi|14277700	RPS12	40S ribosomal protein S12	0.68	0.79	0.70	0
744	gi|4506685	RPS13	40S ribosomal protein S13	1.15*	0.68	0.36	0
745	gi|5032051	RPS14	40S ribosomal protein S14	1.72	1.65	0.91	0
746	gi|4506687	RPS15	40S ribosomal protein S15 isoform 2	2.30	0.99	0.48	0
747	gi|14165469	RPS15A	40S ribosomal protein S15a	0.93	1.02	0.41	0
748	gi|4506691	RPS16	40S ribosomal protein S16	0.85	0.84	0.16*	0
749	gi|4506693	RPS17	40S ribosomal protein S17	1.23	1.01	0.27	0
750	gi|11968182	RPS18	40S ribosomal protein S18	1.51*	1.41	0.41	0
751	gi|4506695	RPS19	40S ribosomal protein S19	1.23	0.93	0.52	0
752	gi|15055539	RPS2	40S ribosomal protein S2	1.28	0.90	0.66	0

753	gi|4506701	RPS23	40S ribosomal protein S23	0.92	1.03	0.49	0
754	gi|4506713	RPS27A	ubiquitin-40S ribosomal protein S27a precursor	1.26	1.05	0.47*	0
755	gi|4506715	RPS28	40S ribosomal protein S28	1.73	0.95	0.24	0
756	gi|15718687	RPS3	40S ribosomal protein S3 isoform 1	0.56	0.64*	1.01	0
757	gi|4506723	RPS3A	40S ribosomal protein S3a isoform 1	1.20	0.71	0.63*	0
758	gi|4506725	RPS4X	40S ribosomal protein S4, X isoform X isoform	0.94	0.80	0.18*	0
759	gi|13904870	RPS5	40S ribosomal protein S5	0.79	1.32	0.60	0
760	gi|17158044	RPS6	40S ribosomal protein S6	1.03	1.32	0.16*	0
761	gi|4506741	RPS7	40S ribosomal protein S7	0.79	0.66	0.58	0
762	gi|4506743	RPS8	40S ribosomal protein S8	0.89	1.12	0.54	0
763	gi|14141193	RPS9	40S ribosomal protein S9	1.00	0.89	0.31*	0
764	gi|9845502	RPSA	40S ribosomal protein SA isoform 1	1.00	1.15	0.37*	0
765	gi|110611218	RRBP1	ribosome-binding protein 1	0.98	1.54*	1.64*	0
766	gi|4506749	RRM1	ribonucleoside-diphosphate reductase large subunit	0.95	0.53*	1.34	0
767	gi|7657015	RTCB	tRNA-splicing ligase RtcB homolog	1.00	0.67*	0.90	0
768	gi|4506753	RUVBL1	ruvB-like 1	1.24	1.07	1.49*	0
769	gi|5730023	RUVBL2	ruvB-like 2	0.99	0.86	2.95*	0
770	gi|5032057	S100A11	protein S100-A11	0.66	0.78	0.85	0
771	gi|5174659	S100A13	protein S100-A13	0.78	0.95	0.74	0
772	gi|4885585	SAE1	SUMO-activating enzyme subunit 1 isoform a	1.99	0.72	0.47	0

773	gi|7661936	SAFB2	scaffold attachment factor B2	1.23	0.95	0.73	2
774	gi|9910542	SAR1A	GTP-binding protein SAR1a	0.70	1.01	1.36	0
775	gi|32129199	SARNP	SAP domain-containing ribonucleoprotein	0.77	0.76	0.3*	0
776	gi|16306548	SARS	serine--tRNA ligase, cytoplasmic	0.84	1.09	0.68	0
777	gi|16445419	SCAMP3	secretory carrier-associated membrane protein 3 isoform 1	0.97	1.04	1.20	2
778	gi|449083351	SEC16A	protein transport protein Sec16A isoform 2	0.94	1.02	0.38	5
779	gi|380837121	SEC22B	vesicle-trafficking protein SEC22b precursor	0.81	0.93	1.11	0
780	gi|38373669	SEC24C	protein transport protein Sec24C	0.74	0.39*	1.00	1
781	gi|385137130	SELENBP1	selenium-binding protein 1 isoform 3	0.56	1.11	0.90	0
782	gi|4758158	SEPT2	septin-2 isoform a	1.05	1.01	0.70	0
783	gi|148352331	SEPT7	septin-7 isoform 1	1.15	1.12	0.68	0
784	gi|13489087	SERPINB1	leukocyte elastase inhibitor	0.75	1.08	0.76	0
785	gi|167860126	SERPINB5	serpin B5	0.73	0.72	0.79	0
786	gi|32454741	SERPINH1	serpin H1 precursor	1.01	1.42*	2.25*	0
787	gi|5032087	SF3A1	splicing factor 3A subunit 1	0.92	1.13	1.8*	10
788	gi|5803167	SF3A3	splicing factor 3A subunit 3	1.21	0.73	1.29	0
789	gi|54112117	SF3B1	splicing factor 3B subunit 1 isoform 1	1.15	1.04	1.09*	1
790	gi|7706326	SF3B14	splicing factor 3B subunit 6	1.29	1.04	0.83	0
791	gi|55749531	SF3B2	splicing factor 3B subunit 2	2.65*	1.03	0.36*	11
792	gi|54112121	SF3B3	splicing factor 3B subunit 3	0.62	0.65*	1.75*	0

793	gi|5454052	SFN	14-3-3 protein sigma	1.30	1.27	1.06	0
794	gi|4826998	SFPQ	splicing factor, proline- and glutamine-rich	1.31*	0.66	1.14	11
795	gi|23618867	SFXN1	sideroflexin-1	0.65	0.31	3.18*	0
796	gi|13775198	SH3BGRL3	SH3 domain-binding glutamic acid-rich-like protein 3	0.82	0.75	2.97	0
797	gi|4506929	SH3GL1	endophilin-A2 isoform 1	0.95	1.46	1.07	0
798	gi|7705773	SH3GLB1	endophilin-B1 isoform 1	1.30	0.79	0.33	0
799	gi|63055059	SH3PXD2B	SH3 and PX domain-containing protein 2B isoform a	1.21	0.99	0.74	2
800	gi|22547189	SHMT1	serine hydroxymethyltransferase, cytosolic isoform 2	0.50	0.53	0.29*	0
801	gi|23397666	SIN3A	paired amphipathic helix protein Sin3a	1.13	0.89	0.99	2
802	gi|25777713	SKP1	S-phase kinase-associated protein 1 isoform b	0.83	0.65	1.18	1
803	gi|5032093	SLC1A5	neutral amino acid transporter B(0) isoform 1	1.43	0.41	1.71	0
804	gi|156071459	SLC25A5	ADP/ATP translocase 2	0.88*	1.10	1.6*	0
805	gi|5902090	SLC2A3	solute carrier family 2, facilitated glucose transporter member 3	1.08	0.95	1.15	0
806	gi|52352803	SLC30A1	zinc transporter 1	1.73	0.81	1.03	0
807	gi|65506891	SLC3A2	4F2 cell-surface antigen heavy chain isoform c	1.24	0.56*	1.67*	0
808	gi|71979932	SLC7A5	large neutral amino acids transporter small subunit 1	0.92	0.77	2.28	0
809	gi|4759140	SLC9A3R1	Na(+)/H(+) exchange regulatory cofactor NHE-RF1	1.03	0.86	0.5*	0
810	gi|41281453	SLK	STE20-like serine/threonine-protein kinase isoform 1	1.31*	0.51*	0.75	0
811	gi|188536047	SMARCC1	SWI/SNF complex subunit SMARCC1	1.07	0.82	1.37	8
812	gi|21264355	SMARCE1	SWI/SNF-related matrix-associated actin-dependent regulator of chromatin subfamily E member 1	0.85	1.19	1.31	1

813	gi|30581135	SMC1A	structural maintenance of chromosomes protein 1A isoform 1	0.91	0.79	1.24	0
814	gi|110347418	SMC2	structural maintenance of chromosomes protein 2	0.75	0.78	0.86	0
815	gi|4885399	SMC3	structural maintenance of chromosomes protein 3	1.45*	0.36	1.01	0
816	gi|109948304	SMU1	WD40 repeat-containing protein SMU1	1.09	2.48	1.76	0
817	gi|209693439	SNCG	gamma-synuclein	0.70	1.17	0.2*	0
818	gi|77404397	SND1	staphylococcal nuclease domain-containing protein 1	0.94	0.68	0.36*	1
819	gi|40217847	SNRNP200	U5 small nuclear ribonucleoprotein 200 kDa helicase	0.78	0.80	1.22*	1
820	gi|4759156	SNRPA	U1 small nuclear ribonucleoprotein A	0.74	1.21	0.77*	2
821	gi|50593002	SNRPA1	U2 small nuclear ribonucleoprotein A'	0.92	0.78	0.39*	0
822	gi|4507123	SNRPB2	U2 small nuclear ribonucleoprotein B"	0.90	0.91	0.78	0
823	gi|5902102	SNRPD1	small nuclear ribonucleoprotein Sm D1 isoform 1	0.75	1.21	0.36	0
824	gi|4759158	SNRPD2	small nuclear ribonucleoprotein Sm D2 isoform 1	0.47*	0.87	0.53	0
825	gi|4759160	SNRPD3	small nuclear ribonucleoprotein Sm D3	0.89	0.94	0.21	0
826	gi|4507133	SNRPG	small nuclear ribonucleoprotein G	2.41	1.04	0.41	0
827	gi|6912676	SNW1	SNW domain-containing protein 1	1.03	0.97	1.03	1
828	gi|23111038	SNX2	sorting nexin-2 isoform 1	0.70	0.56	0.52	1
829	gi|156627571	SORD	sorbitol dehydrogenase	1.31	0.84	0.68	0
830	gi|27436920	SPAG9	C-Jun-amino-terminal kinase-interacting protein 4 isoform 3	0.70	0.76	0.61	0
831	gi|4507185	SPR	sepiapterin reductase	1.48	0.51	0.94	0
832	gi|112382250	SPTBN1	spectrin beta chain, non-erythrocytic 1 isoform 1	1.04	0.82	0.66	0

833	gi|5902122	SPTBN2	spectrin beta chain, non-erythrocytic 2	0.89	0.7*	0.43	1
834	gi|116089325	SREK1	splicing regulatory glutamine/lysine-rich protein 1 isoform a	0.90	1.07	1.06	0
835	gi|149999611	SRP14	signal recognition particle 14 kDa protein isoform 1	1.69	1.14	0.21*	0
836	gi|4507215	SRP54	signal recognition particle 54 kDa protein isoform 1	1.00	1.02	0.87	0
837	gi|109638749	SRP72	signal recognition particle subunit SRP72 isoform 1	0.55	0.88	1.15*	0
838	gi|118572613	SRRM2	serine/arginine repetitive matrix protein 2	1.23*	0.89	1.04	3
839	gi|5902076	SRSF1	serine/arginine-rich splicing factor 1 isoform 1	0.55	0.72	0.85	0
840	gi|4759100	SRSF11	serine/arginine-rich splicing factor 11 isoform 1	1.41	1.25	0.32	0
841	gi|4506901	SRSF3	serine/arginine-rich splicing factor 3	2.41	1.02	0.45	2
842	gi|86991438	SRSF5	serine/arginine-rich splicing factor 5	0.91	1.13	0.54	0
843	gi|72534660	SRSF7	serine/arginine-rich splicing factor 7 isoform 1	0.85	0.97	0.66	0
844	gi|10835067	SSB	lupus La protein	0.62*	0.71	0.27*	0
845	gi|4507231	SSBP1	single-stranded DNA-binding protein, mitochondrial precursor	1.05	1.38	1.82*	0
846	gi|5454090	SSR4	translocon-associated protein subunit delta isoform 2 precursor	0.74	0.93	2.55	0
847	gi|4507241	SSRP1	FACT complex subunit SSRP1	0.76	0.79	0.70	0
848	gi|19923193	ST13æ	hsc70-interacting protein isoform 1	0.94	1.00	0.21*	0
849	gi|5803181	STIP1	stress-induced-phosphoprotein 1 isoform b	1.40	1.42	0.32*	2
850	gi|5031851	STMN1	stathmin isoform a	0.79	1*	0.34*	0
851	gi|7305503	STOML2	stomatin-like protein 2, mitochondrial isoform a	0.62	1.05	2.95*	0
852	gi|148727341	STRAP	serine-threonine kinase receptor-associated protein	1.00	1.04	0.24*	0

853	gi|56181387	STUB1	E3 ubiquitin-protein ligase CHIP isoform a	0.46*	0.68	0.58	0
854	gi|217330646	SUB1	activated RNA polymerase II transcriptional coactivator p15	1.74	1.47	0.36*	0
855	gi|6005757	SUPT16H	FACT complex subunit SPT16	0.59	0.65	0.47	0
856	gi|19557702	SURF6	surfeit locus protein 6 isoform 1	0.92	0.72	0.59	0
857	gi|5803187	TALDO1	transaldolase	1.31	1.51	0.41	0
858	gi|6678271	TARDBP	TAR DNA-binding protein 43	0.91	0.64	0.56	0
859	gi|38202255	TARS	threonine--tRNA ligase, cytoplasmic isoform 1	0.78	0.86	0.23*	0
860	gi|4759212	TBCA	tubulin-specific chaperone A isoform 2	1.25	1.24	0.28*	0
861	gi|5032161	TCEB1	transcription elongation factor B polypeptide 1 isoform a	1.05	0.64	0.58	0
862	gi|6005890	TCEB2	transcription elongation factor B polypeptide 2 isoform a	0.69	2.15*	0.29*	0
863	gi|91208418	TCERG1	transcription elongation regulator 1 isoform 2	0.85	1.10	0.45*	9
864	gi|207113160	TCOF1	treacle protein isoform d	1.24	1.17	0.75	0
865	gi|57863257	TCP1	T-complex protein 1 subunit alpha isoform a	0.96	0.88	1.16	0
866	gi|7661666	TES	testin isoform 1	0.71	0.32	0.51	0
867	gi|4507401	TFAM	transcription factor A, mitochondrial isoform 1 precursor	0.96	1.55	1.88*	0
868	gi|306774089	TFG	protein TFG isoform 2	0.90	0.85	0.69	1
869	gi|189458817	TFRC	transferrin receptor protein 1	0.82	0.55	1.81*	0
870	gi|4507491	THOP1	thimet oligopeptidase	0.60	1.00	0.3*	0
871	gi|167234419	THRAP3	thyroid hormone receptor-associated protein 3	0.90	0.84	0.42*	1
872	gi|9966849	TIGAR	fructose-2,6-bisphosphatase TIGAR	1.19	1.01	0.35	0

873	gi|4758152	TIMM8A	mitochondrial import inner membrane translocase subunit Tim8 A isoform 1	1.05	1.01	1.13	0
874	gi|4507521	TKT	transketolase isoform 1	1.02	0.86*	0.25*	0
875	gi|223029410	TLN1	talin-1	1.18*	1.82*	1.34	0
876	gi|98986464	TMED10	transmembrane emp24 domain-containing protein 10 precursor	1.52*	1.03	2.17*	0
877	gi|7657649	TMOD3	tropomodulin-3	1.04	1.92	2.07*	0
878	gi|4507555	TMPO-a	thymopoietin isoform alpha	0.86	1.10	1.21	0
879	gi|73760405	TMPO-b	thymopoietin isoform beta	0.56	0.69	0.89	0
880	gi|10863895	TMSB10	thymosin beta-10	1.94	1.34	0.19	0
881	gi|11056061	TMSB4X	thymosin beta-4	3.27	3.16*	0.31*	0
882	gi|110556636	TNKS1BP1	182 kDa tankyrase-1-binding protein	1.24	1.11	0.61	5
883	gi|23510381	TNPO1	transportin-1 isoform 2	0.50	0.52	1.16	0
884	gi|21361356	TOMM34	mitochondrial import receptor subunit TOM34	1.13	1.03	0.67	0
885	gi|54607135	TOMM70A	mitochondrial import receptor subunit TOM70	0.82	1.43	1.86*	0
886	gi|389886539	TOR1AIP1	torsin-1A-interacting protein 1 isoform 1	1.36	0.78	0.81	0
887	gi|5032189	TP53BP1	tumor suppressor p53-binding protein 1 isoform 3	2.54*	1.69*	1.47	1
888	gi|70608174	TPD52	tumor protein D52 isoform 2	1.00	0.75	0.39	0
889	gi|226529917	TPI1	triosephosphate isomerase isoform 2	1.02	1.40	0.15*	0
890	gi|63252900	TPM1	tropomyosin alpha-1 chain isoform Tpm1.7cy	1.17	1.38	0.98	0
891	gi|4507651	TPM4	tropomyosin alpha-4 chain isoform Tpm4.2cy	0.93	2.52*	0.69	0
892	gi|186972143	TPP2	tripeptidyl-peptidase 2	1.46	1.37	0.70	1

893	gi|114155142	TPR	nucleoprotein TPR	0.83	1.67*	1.59*	0
894	gi|4759098	TRA2B	transformer-2 protein homolog beta isoform 1	0.84	0.87	0.71	0
895	gi|68160937	TRIM25	E3 ubiquitin/ISG15 ligase TRIM25	1.03	0.47	0.16	1
896	gi|5032179	TRIM28	transcription intermediary factor 1-beta	0.95	0.76*	0.15*	1
897	gi|54792146	TRIM47	tripartite motif-containing protein 47	1.21	0.82	0.48	0
898	gi|11342676	TRIP10	cdc42-interacting protein 4 isoform 2	0.55	1.05	0.48	0
899	gi|7705477	TRMT112	multifunctional methyltransferase subunit TRM112-like protein isoform 1	0.67	0.63*	0.15*	0
900	gi|31377800	TROVE2	60 kDa SS-A/Ro ribonucleoprotein isoform 2	1.22	0.72	0.79	0
901	gi|5174731	TSNAX	translin-associated protein X	1.36	0.88	0.47	0
902	gi|17402865	TST	thiosulfate sulfurtransferase	1.63	0.91	0.50	0
903	gi|11056036	TTLL12	tubulin--tyrosine ligase-like protein 12	1.72	0.85	0.44*	1
904	gi|14389309	TUBA1C	tubulin alpha-1C chain isoform c	1.08	0.87	0.57	0
905	gi|17921989	TUBA4A	tubulin alpha-4A chain isoform 1	1.52	0.82	0.25	0
906	gi|29788785	TUBB	tubulin beta chain isoform b	1.69	0.61	0.49	0
907	gi|4507729	TUBB2A	tubulin beta-2A chain isoform 1	0.87	1.00	1.06	0
908	gi|5174735	TUBB4B	tubulin beta-4B chain	0.94	0.90	0.41	0
909	gi|14210536	TUBB6	tubulin beta-6 chain isoform 1	1.27	0.83	0.7*	0
910	gi|34147630	TUFM	elongation factor Tu, mitochondrial precursor	1.19	0.61	0.1*	0
911	gi|6005846	TWF2	twinfilin-2	1.16	1.03	1.17	0
912	gi|50592994	TXN	thioredoxin isoform 1	0.55	1.13	0.16*	0

913	gi|14249348	TXNDC17	thioredoxin domain-containing protein 17	0.83	1.68	0.24	0
914	gi|42794771	TXNDC5	thioredoxin domain-containing protein 5 isoform 1 precursor	1.55*	2.42*	2.15*	0
915	gi|4759274	TXNL1	thioredoxin-like protein 1	0.98	1.01	0.56	0
916	gi|4503445	TYMP	thymidine phosphorylase isoform 1 proprotein	0.77	0.38	0.27*	0
917	gi|60279268	U2AF2	splicing factor U2AF 65 kDa subunit isoform b	1.24	0.45*	0.16*	1
918	gi|23510338	UBA1	ubiquitin-like modifier-activating enzyme 1	1.24	0.91	0.51	0
919	gi|150417996	UBA6	ubiquitin-like modifier-activating enzyme 6	0.89	0.82	0.80	0
920	gi|4507791	UBE2M	NEDD8-conjugating enzyme Ubc12	1.54	0.44*	0.75	0
921	gi|4507793	UBE2N	ubiquitin-conjugating enzyme E2 N	1.16	1.25	0.16	0
922	gi|16753203	UBQLN1	ubiquilin-1 isoform 1	0.95	0.85	1.40	0
923	gi|40538799	UBQLN4	ubiquilin-4 isoform 1	1.26	0.95	1.15	0
924	gi|82659109	UBR4	E3 ubiquitin-protein ligase UBR4	0.83	0.48	1.62	4
925	gi|15147337	UBR5	E3 ubiquitin-protein ligase UBR5 isoform 1	0.58	0.68	0.77	1
926	gi|21361517	UBXN1	UBX domain-containing protein 1 isoform 1	0.68	0.96	0.45	1
927	gi|4507813	UGDH	UDP-glucose 6-dehydrogenase isoform 1	0.92	1.02*	1.02	0
928	gi|9910280	UGGT1	UDP-glucose:glycoprotein glucosyltransferase 1 precursor	0.96	0.76*	2.75*	1
929	gi|89179321	UNC45A	protein unc-45 homolog A isoform 3	0.90	1.18	0.56	0
930	gi|18375673	UPF1	regulator of nonsense transcripts 1 isoform 2	0.99	1.11	1.07	1
931	gi|5454152	UQCRB	cytochrome b-c1 complex subunit 7 isoform 1	0.69	0.85	4.09	0
932	gi|46593007	UQCRC1	cytochrome b-c1 complex subunit 1, mitochondrial precursor	1.08*	1.05	1.9*	0

933	gi|50592988	UQCRC2	cytochrome b-c1 complex subunit 2, mitochondrial precursor	0.64	1.01	2.64*	0
934	gi|163644321	UQCRFS1	cytochrome b-c1 complex subunit Rieske, mitochondrial	1.09	0.71	3.77*	0
935	gi|71051616	UROD	uroporphyrinogen decarboxylase	2.18	1.06	0.37	0
936	gi|4827050	USP14	ubiquitin carboxyl-terminal hydrolase 14 isoform a	0.81	0.28*	0.30	0
937	gi|150378533	USP7	ubiquitin carboxyl-terminal hydrolase 7 isoform 1	0.97	0.80	1.20	0
938	gi|21361348	UTP14A	U3 small nucleolar RNA-associated protein 14 homolog A isoform 1	1.68	0.39	0.28	0
939	gi|5454158	VARS	valine--tRNA ligase	0.78	0.61	0.37	5
940	gi|18379349	VAT1	synaptic vesicle membrane protein VAT-1 homolog	1.17	1.37	0.61	1
941	gi|4507873	VBP1	prefoldin subunit 3 isoform 1	0.94	1.25	1.25	0
942	gi|6005942	VCP	transitional endoplasmic reticulum ATPase	1.36	1.49*	0.85	0
943	gi|4507879	VDAC1	voltage-dependent anion-selective channel protein 1	0.67*	0.92	2.18*	0
944	gi|25188179	VDAC3	voltage-dependent anion-selective channel protein 3 isoform 1	0.99	0.71	1.54	0
945	gi|62414289	VIM	vimentin	1.63*	1.21*	1.15	0
946	gi|7706441	VPS29	vacuolar protein sorting-associated protein 29 isoform 1	0.92	0.81	0.59	0
947	gi|17999541	VPS35	vacuolar protein sorting-associated protein 35	0.80	0.43	1.24	0
948	gi|47419914	WARS	tryptophan--tRNA ligase, cytoplasmic isoform a	2.00	0.80	0.36*	0
949	gi|9257257	WDR1	WD repeat-containing protein 1 isoform 1	0.81	0.96	0.37*	0
950	gi|56243583	WDR18	WD repeat-containing protein 18	1.12	0.93	1.69	0
951	gi|45238858	WDR44	WD repeat-containing protein 44 isoform 1	0.76	1.35	0.96	4
952	gi|38327642	WDR55	WD repeat-containing protein 55	1.11	1.18	0.76	0

953	gi|13376840	WDR61	WD repeat-containing protein 61 isoform a	1.18	1.17	1.01	0
954	gi|221219004	WDR74	WD repeat-containing protein 74 isoform 1	1.03	0.94	0.82	0
955	gi|13129110	WDR77	methylosome protein 50	0.75	1.06	0.95	1
956	gi|264681563	XPNPEP1	xaa-Pro aminopeptidase 1 isoform 1	0.84	0.78	0.48*	0
957	gi|11559925	XPNPEP3	probable Xaa-Pro aminopeptidase 3 isoform 1	1.23	0.65	1.63	0
958	gi|4507943	XPO1	exportin-1	1.03	0.74	0.88	0
959	gi|10863945	XRCC5	X-ray repair cross-complementing protein 5	0.77	0.68	0.98	0
960	gi|4503841	XRCC6	X-ray repair cross-complementing protein 6 isoform 1	0.81	0.94	0.80	1
961	gi|18860916	XRN2	5'-3' exoribonuclease 2	0.98	0.74	1.12	3
962	gi|4507947	YARS	tyrosine--tRNA ligase, cytoplasmic	1.06	0.68	0.41	0
963	gi|34098946	YBX1	nuclease-sensitive element-binding protein 1	1.10	1.01	0.63	0
964	gi|4507949	YWHAB	14-3-3 protein beta/alpha	0.66	0.72	0.44*	0
965	gi|5803225	YWHAE	14-3-3 protein epsilon	0.97	0.92*	0.37*	0
966	gi|21464101	YWHAG	14-3-3 protein gamma	0.53	0.92	0.52	0
967	gi|4507951	YWHAH	14-3-3 protein eta	1.06	1.36	0.29	0
968	gi|5803227	YWHAQ	14-3-3 protein theta	1.95	1.43	0.62	0
969	gi|4507953	YWHAZ	14-3-3 protein zeta/delta	0.90	0.80	0.22*	0
970	gi|126723060	ZC3H4	zinc finger CCCH domain-containing protein 4	0.68	1.44	0.93	11
971	gi|27477136	ZC3HAV1	zinc finger CCCH-type antiviral protein 1 isoform 1	1.13	1.03	0.88	1
972	gi|4508047	ZYX	zyxin	1.72	2.1*	0.65	9
